# Recent Progress in Flexible Organic Thermoelectrics

**DOI:** 10.3390/mi9120638

**Published:** 2018-11-30

**Authors:** Mario Culebras, Kyungwho Choi, Chungyeon Cho

**Affiliations:** 1 Stokes Laboratories, Bernal Institute, University of Limerick, Limerick, Ireland; mario.culebrasrubio@ul.ie; 2 Transportation Innovative Research Center, Korea Railroad Research Institute, Uiwang-si 16105, Korea; kwchoi80@krri.re.kr; 3 Department of Carbon Convergence Engineering, College of Engineering, Wonkwang University, Iksan 54538, Korea

**Keywords:** thermoelectric, graphene, carbon nanotubes, power factor, polymers, energy harvesting, organic composites

## Abstract

Environmental energy issues caused by the burning of fossil fuel such as coal, and petroleum, and the limited resources along with the increasing world population pose a world-wide challenge. Alternative energy sources including solar energy, wind energy, and biomass energy, have been suggested as practical and affordable solutions to future energy needs. Among energy conversion technologies, thermoelectric (TE) materials are considered one of the most potential candidates to play a crucial role in addressing today’s global energy issues. TE materials can convert waste heat such as the sun, automotive exhaust, and industrial processes to a useful electrical voltage with no moving parts, no hazardous working chemical-fluids, low maintenance costs, and high reliability. These advantages of TE conversion provide solutions to solve the energy crisis. Here, we provide a comprehensive review of the recent progress on organic TE materials, focused on polymers and their corresponding organic composites incorporated with carbon nanofillers (including graphene and carbon nanotubes). Various strategies to enhance the TE properties, such as electrical conductivity and the Seebeck coefficient, in polymers and polymer composites will be highlighted. Then, a discussion on polymer composite based TE devices is summarized. Finally, brief conclusions and outlooks for future research efforts are presented.

## 1. Introduction

The global demand for sustainable and renewable energy sources due to climate change caused by burning fossil fuels has incurred interest in developing new types of energy [1]. Various renewable resources including solar energy, wind, geothermal, and wave have been investigated to produce power without giving rise to any carbon dioxide emissions, but their performance has still been limited [2,3]. Considering that more than 60% of global energy is produced by cars, transportation, and mechanical systems is wasted as the form of heat, reconverting this wasted heat into useful electricity can be a viable way in addressing current global energy issues [4,5,6]. The direct conversion between waste heat and electrical power based on thermoelectric (TE) materials has attracted tremendous attention because they have various applications such as eco-friendly power generation and cooling systems with no moving parts and pollution-free step.

The efficiency of TE materials is evaluated by a dimensionless figure-of-merit, ZT, which is defined as S^2^∙σ∙T∙k^−1^, where S, σ, κ, and T are the Seebeck coefficient, electrical conductivity, thermal conductivity, and absolute temperature of the material, respectively. The power factor (PF), defined as S^2^∙σ, is occasionally used as an alternative to ZT because of the low κ for polymer-based TE materials or difficulties in the precise in-plane κ measurement for thin film thickness (<1 μm) [7,8]. A high-performance TE material should possess a high S to promote the energy conversion of heat to electricity, a high σ to minimize Joule heating, and a low κ to keep a temperature gradient [9]. However, it is very difficult to modulate independently these three parameters because of a strong interdependence between them in traditional bulk materials, which imposes a limitation on maximizing ZT as a whole. For example, increasing σ is usually accompanied by a decreased S and an increased carrier contribution to κ.

Recent advances in engineering the electronic structure of TE nanostructures have led to a significant improvement of ZT of >1 by preferentially reducing the lattice κ by involving the phonon scattering effect within superlattices or nanoinclusion [10]. Moreover, the energy filtering effect, quantum confinement, and tuning the electronic band structure (i.e., the density of the states) through nanostructures engineering have been shown to independently improve S without greatly suppressing σ (hence, the enhancement of PF) [11,12,13]. When the system size approaches a scale comparable to the feature length of electron behavior (i.e., mean free path, wavelength) in any direction, low-dimensional materials could create the spike-like shape of electronic density of states (e.g., Van Hove singularities), which could result in increased asymmetry of the differential conductivity at around the Fermi energy $\left[\frac{\partial \mathrm{DOS}\;\left(E\right)}{\partial E}\right]$_E = EF_ [11].

Currently, the most widely used TE materials are conventional inorganic semiconductors based on metal alloys (i.e., Bi_2_Te_3_, SiGe, and PbTe) due to their high PF [14]. Besides their high TE efficiency, the inorganic-based TE materials have been used to power space vehicles because of their suitability for high-temperature (600–1000 °C) power generation [15]. Despite their promise, they possess certain inherent limitations such as a scarcity of materials, high cost of production, brittleness, the difficulty of processing, and toxicity that hinder the full utilization of their unique benefits. Organic materials could alleviate these problems because of their advantageous properties, including having a light weight, low cost, mechanical flexibility, convenience to be processed, and solution processability over large areas [16,17]. Moreover, compared to semiconductor-based TE materials, organic materials possess an intrinsically low κ, typically ranging from 0.1 to 1 W∙m^−1^∙K^−1^, which is beneficial to the enhancement of TE performance [8,18]. Although there has been progress, the PF of conducting polymers is low relative to inorganic materials, and their ZT is 2–3 orders of magnitude lower than that of the inorganic TE materials because of their low σ and S, which hampers their use in TE applications [19]. Recently, organic TE nanocomposites have been exhibited to achieve high TE performance by judicious combination with low-dimensional carbon nanofillers such as graphene or carbon nanotubes (CNTs). Incorporating carbon materials into polymer matrices results in synergies, such as a high σ and S, while retaining low κ [20].

In this review, the recent advances in the TE performances of polymers and their corresponding organic composites comprised of carbon nanofillers, including graphene, graphene oxide, and carbon nanotubes, are highlighted. We discuss the preparation strategies to optimize the TE properties and organic TE devices are also summarized. Finally, a brief conclusion has been provided along with a perspective and outlook on the future development of flexible organic TE materials.

## 2. Polymer-Based Thermoelectric Materials

Since the discovery of iodine-doped polyacetylene that conducts like a metal by MacDiarmid [21], intrinsically conducting polymers have been widely explored for various applications in the area of optoelectronic devices, supercapacitors, biosensors, photovoltaic cells, batteries and electromagnetic shielding [22,23]. This is related to the set of advantageous properties associated with their unique structures with excellent physical and chemical properties [24]. Among the various conducting polymers, considerable attention has been recently paid to polyaniline (PANi) and poly(3,4-ethylenedioxythiophene)-poly(styrenesulfonate) (PEDOT:PSS), Poly(3-hexylthiophene) (P3HT), and their derivatives as promising candidates of TE materials. The chemical structure of intrinsically conductive polymers, along with the insulating polymers discussed in this review, is shown in Table 1. A summary of the TE properties of polymers and their composites compounded with carbon nanofillers is provided in Table 2. In this section, we have highlighted the TE performance of conducting polymers such as PANi, PEDOT and its derivatives, and P3HT.

### 2.1. Polyaniline (PANi)

Polyaniline (PANi) has been considered one of the most promising conductive polymers with merits of the structure diversification, environmental stability, and unique doping/dedoping process. The very first investigation on the TE properties of PANi was done by Yan and Toshima in 1999 [26]. They fabricated multilayered thin films by alternately layering electrically insulating emeraldine base layers and electrically conducting (±)-10-camphorsulfonic acid (CSA)-doped emeraldine salt layers. By doping the films with CSA, multilayers consisting of the two kinds of PANi exhibited an electrical conductivity of 173 S∙cm^−1^ and Seebeck coefficient of 14 μV∙K^−1^, translating to the power factor of 3.5 μW∙m^−1^∙K^−2^ at 300 K. This value was 3.5 times higher relative to that of the bulk counterpart. The same research group also investigated the anisotropy of the TE properties in CSA-doped PANi films [27]. In their study, the PANi molecules took an ordered and extended coil-like conformation upon the stretching process, resulting in enhancement in carrier mobility and hence, electrical conductivity and Seebeck coefficient in the PANi films. The stretched CSA-doped PANi films exhibited higher values in ZT (2.9 × 10^−2^) compared to that of the unstretched counterpart (4.4 × 10^−3^).

The TE properties of PANi can be modulated by controlling its structure [84]. The submicron-fiber structure of PANi was created by doping with CSA and followed by mixing with m-cresol. PANi(CSA)/m-cresol had a great quantity of submicron fibers with a typical size of 200–500 nm in diameter and 5–10 μm in length. PANi(CSA) samples with no m-cresol treatment displayed a general grain structure. PANi(CSA)/m-cresol exhibited the electrical conductivity of 67 S∙cm^−1^ and the Seebeck coefficient of 12.8 μV∙K^−1^. This resulted in a power factor of 1.1 μW∙m^−1^∙K^−2^ at 300 K, which was about 20 times higher than that of PANi(CSA). The higher TE properties in PANi(CSA)/m-cresol over PANi(CSA) were due to structural changes of PANi that CSA-doped PANi formed liquid-crystalline solutions in m-cresol with an extraordinary order of chain packing [85], which was confirmed with X-ray diffraction (XRD) patterns. Therefore, the submicron-fiber structure where the oriented PANi chains were clustered had less π-conjugation defects in the polymer backbone, which was beneficial for improving the carrier mobility and, hence, the electrical conductivity.

Li et al. studied the effects of hydrochloric acid (HCl)-doping concentrations on the TE properties of PANi [29]. HCl-doped PANi films prepared by chemical oxidative polymerization exhibited that elevated HCl-doping levels led to an increase in electrical conductivity up to 600 S∙cm^−1^ at 1.25 M and then a dramatic reduction above this concentration. The Seebeck coefficient showed an opposite trend to that of the electrical conductivity in which a maximum value was obtained at 0.25 M. Due to its mainly amorphous structure, the thermal conductivity of PANi was in the range of 0.1 W∙m^−1^∙K^−1^ [86]. In this work, the thermal conductivity was insensitive to doping conditions, showing 0.276 W∙m^−1^∙K^−1^ at 303 K. The maximum ZT reached 2.7 × 10^−4^ at 423 K when the HCl-doping concentration was 1.0 M.

**Figure 1:**
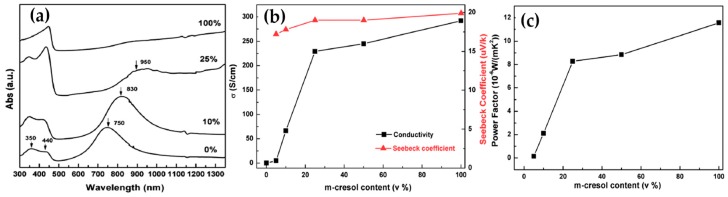
(**a**) The ultraviolet-visible (UV-vis) spectra of polyaniline (PANi) dissolved in different m-cresol contents, (**b**) the electrical conductivity and Seebeck coefficient, and (**c**) the power factor of the polyaniline films prepared with different m-cresol contents. Reprinted with permission from [28]. Copyright 2014 The Royal Society of Chemistry.

Modulating the ordered degree of polymeric molecular chains is an effective way to realize the synergistic regulation of electrical conductivity and Seebeck coefficient and therefore improve the TE properties of conducting polymers [28]. Yao et al. investigated the intrinsic effect of the molecular structure on the electric transport of PANi by tuning the molecular chain alignment. The strong van der Waals attractions between polymer chains in most solvents caused PANi to adopt a compacted coil conformation. However, the chemical interactions between CSA-doped PANi and m-cresol induced the molecular structure of PANi to change to an expanded coil, which was confirmed with the ultraviolet-visible (UV-vis) spectra in Figure 1a. This structural change of PANi to the expanded molecular conformation reduced the π defects caused by ring twisting and strengthened the π-π conjugation interactions between rings and therefore enhanced the delocalization of carriers along the polymer chain. The ordered chain packing increased the carrier mobility and improved both the electrical conductivity and Seebeck coefficient (and hence, the power factor) with the m-cresol content, as shown in Figure 1b,c.

### 2.2. Poly(3,4-ethylenedioxythiophene) (PEDOT) and Their Derivatives

The electrically conductive polymer PEDOT, one of the polythiophene derivatives, has gained particular attention due to their highly conductive properties, optical transparency, solution processability, and remarkable capabilities of easy doping [87]. The highly electro-conducting PEDOT films have been used as effective organic materials for many applications including organic solar cells, light emitting diodes, and organic field-effect transistors. The TE properties of PEDOT have been shown to be controlled precisely via chemical or electrochemical doping that enhances electrical conductivity without significantly compromising the Seebeck coefficient, thus achieving the improved TE properties. For instance, Bubnova et al. demonstrated the optimization of ZT in PEDOT:p-toluenesulfonate (PEDOT:Tos) by using tetrakis(dimethylamino)ethylene (TDAE) as the de-doping agent [88]. Upon exposure of a PEDOT:Tos film into a vapor of TDAE molecules, electron transfer from the reducing agent to the polymer transformed oxidized PEDOT:Tos chains into neutral ones. At the optimum oxidation level of 22%, they reported a very high power factor of 324 μW∙m^−1^∙K^−2^ and a low thermal conductivity of 0.37 W∙m^−1^∙K^−2^, yielding a ZT = 0.25 at room temperature. Another high TE performance of PEDOT films was prepared through the precise control of the oxidation level of the polymer electrochemically [89]. A mixture of pyridine and PEG-b-poly(propylene glycol)-b-PEG tri-block copolymer was used as mediators for the polymerization of 3,4-ethylenedioxythiophene (EDOT) in the presence of Fe-tosylate (Tos). A maximum power factor of 1270 μW∙m^−1^∙K^−2^ was obtained when the applied potential was 0.1 V.

Of the various PEDOTs available, doping with PSS is the most studied PEDOT derivative because of its inherent properties such as water-solubility, low cost, flexible mechanical properties, thermal stability, high transparency, and commercial availability, making it suitable for TE applications. Although the electrical conductivity of PEDOT:PSS has been shown to be enhanced by a variety of organic solvents such as dimethyl sulfoxide (DMSO), N,N-dimethylformamide (DMF), and tetrahydrofuran (THF), the reported ZT values were as low as 10^−4^ to 10^−2^ [90,91]. However, Kim et al. reported a maximum ZT value of 0.42 by mixing PEDOT:PSS with 5% of DMSO and ethylene glycol (EG) in 2013 [30]. The incorporation of 0.2 wt% of PEDOT nanowires into PEDOT-based polymer hosts by in situ polymerization led to a power factor as high as 446.6 μW∙m^−1^∙K^−2^ and the ZT value was up to 0.44 at room temperature [92].

Thermoelectric performance of PEDOT:PSS can be enhanced by treatment with secondary dopants. Free-standing flexible and smooth PEDOT:PSS buckypapers, prepared using vacuum-assisted filtration, revealed that the electrical conductivity was enhanced to 1900 S∙cm^−1^ by treating PEDOT:PSS with formic acid [31]. Since the secondary dopants did not change the oxidation level of PEDOT [93], the Seebeck coefficient remained relatively constant. The power factor of the formic acid-treated films was up to 80.6 µW∙m^−1^∙K^−2^ and the thermal conductivity, measured with the Harman method, was 0.2 W∙m^−1^∙K^−1^, which translated to a ZT value as high as 0.32 at room temperature. The enhancement of TE properties in the PEDOT:PSS films was mainly due to the selective removal of PSS chains by secondary dopants with high dielectric constants. The coulombic interaction between the positively charged PEDOT and negatively charged PSS chains were reduced through screening effect of formic acid, which in turn facilitated the removal of non-conductive PSS and led to a three-dimensional conjugated network of highly conjugated PEDOT [94].

**Figure 2:**
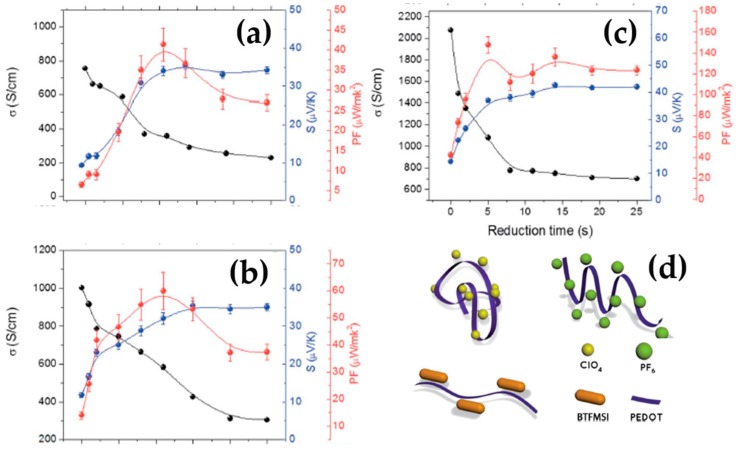
The thermoelectric properties of poly(3,4-ethylenedioxythiophene) (PEDOT):ClO_4_ (**a**), PEDOT:PF_6_ (**b**), and PEDOT: bis(tifluoromethylsulfonyl)imide (BTFMSI) (**c**) as a function of chemical reduction time using hydrazine. PEDOT conformation in the presence of different counter-ions (**d**). Reprinted with permission from [95]. Copyright 2014 The Royal Society of Chemistry.

The TE properties of PEDOT can be finely controlled by doping it with various counter-ions. Culebras et al. investigated the TE performances of different PEDOT derivatives including PEDOT:ClO_4_, PEDOT:PF_6_, and PEDOT:bis(tifluoromethylsulfonyl)imide (BTFMSI), which were synthesized via electro-polymerization [95]. The electrical conductivity was sensitive to the type of dopants, while the Seebeck coefficient remained at the same order of magnitude (Figure 2a–c). The larger size of the counter-ion was more favorable for PEDOT polymers to change from the typical coil conformation to linear or expanded-coil polymeric structures due to the electrostatic interaction between the positive charges of PEDOT and negative charges of counter-ions, as shown in Figure 2d. Therefore, by increasing the size of the counter-ion, the electrical conductivity increased in the order of PEDOT:BTFMSI, PEDOT:PF_6_, and PEDOT:ClO_4_. The TE properties of PEDOT derivatives were further optimized upon the chemical reduction with hydrazine. By reducing them with hydrazine for only 5 s, PEDOT:BTFMSI films exhibited a power factor of 147 µW∙m^−1^∙K^−2^ and a thermal conductivity of 0.19 W∙m^−1^∙K^−1^, which translated to a ZT of 0.22 at room temperature.

Wang et al. reported the effect of the shear printing parameters on the electric transport mechanism in PEDOT films via the natural brush-printing method [96]. They investigated the interplay between backbone alignment, aggregation, and charge transport anisotropy in semiconducting polymers. It was found that the shear-induced charge transport is closely related to the conformational changes of polymer backbones, aggregation, and crystallization during the film formation process. The imposed shear stress enhanced polymer chain alignment, thereby facilitating aggregation and improving charge transport.

**Figure 3:**
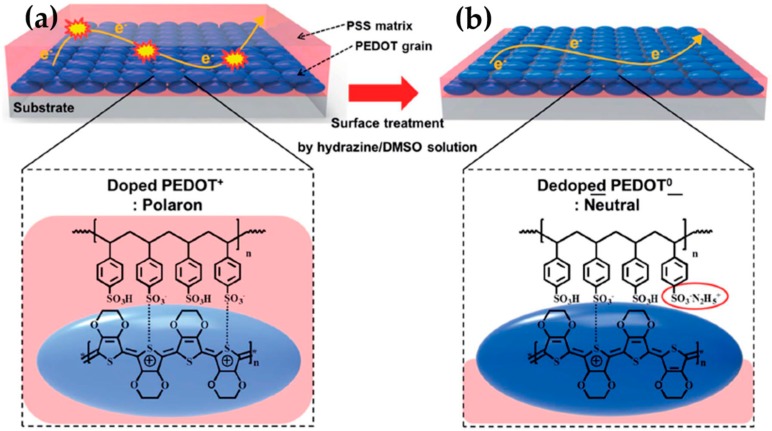
An overall scheme for the spin-coated PEDOT:poly(styrenesulfonate) (PSS) film fabricated by the sequential treatment of p-toluenesulfonic acid monohydrate (TSA)/dimethyl sulfoxide (DMSO) doping and hydrazine/DMSO dedoping. (**a**) DMSO/TSA-doped PEDOT:PSS film formation via spin coating and selective removal of PSS and the dedoped PEDOT:PSS (DDTP) film treated with hydrazine/DMSO solution (**b**). Reprinted with permission from [32]. Copyright 2014 The Royal Society of Chemistry.

Optimizing the oxidation level of PEDOT:PSS films via sequential doping and dedoping can be a promising route toward high TE systems. Lee et al. reported that the synergistic effect of sequential doping with TSA and followed by dedoping with hydrazine/DMSO is an effective way to improve the TE properties by precisely controlling the PSS concentration and the oxidation level of PEDOT. In their study, the treatment of the chemical dopant p-toluenesulfonic acid monohydrate (TSA) on PEDOT:PSS films drove the positively charged holes to more effectively move through the polymer and to facilitate the formation of polarons, consequently increasing the carrier concentration, as shown in Figure 3a [32]. The Coulombic attraction between PEDOT and PSS was weakened because protonated hydrazine ions (N_2_H_5_^+^) were associated with negatively charged PSS (Figure 3b). Furthermore, the dedoping process with the hydrazine/DMSO treatment during spin coating selectively removed insulating PSS molecules in the films. Although the hydrazine/DMSO dedoping decreased the electrical conductivity due to a reduction in the carrier concentration, the Seebeck coefficient dramatically increased. As a result, a power factor was up to 318.4 µW∙m^−1^∙K^−2^ and the thermal conductivity decreased from 0.38 to 0.30 upon the removal of PSS, which translated to a ZT value of 0.31 at room temperature.

A post-treatment with various reagents has been shown to enhance the TE performance. Zhu et al. found out that the TE properties of PEDOT:PSS films were enhanced by employing a deep eutectic solvent (DES), which is a new generation of green solvents having merits such as biocompatibility, non-toxicity, biodegradability, and chemical inertness with water [97,98]. After dropping DES, a mixture of choline chloride and ethylene glycol in the ratio of 1:3, onto PEDOT:PSS prepared by the casting method, the PEDOT:PSS films exhibited an improvement in electrical conductivity to 620 S∙cm^−1^ and in the Seebeck coefficient to 29.1 µV· K^−1^. At an optimized condition (at 100 °C for 10 h heating) of DES treatment, the power factor reached 24 µW∙m^−1^∙K^−2^, which was approximately four orders of magnitude higher relative to the pure PEDOT:PSS. Atomic force microscope (AFM) and X-ray photoelectron spectroscopy (XPS) measurements revealed that the remarkably enhanced electrical conductivity originated from the removal of the excess insulating PSS and the phase separation between the PEDOT and PSS chains. The removal of the excess insulating PSS resulted in a continuous conducting network of the PEDOT-rich phase, which in turn improved the electrical conductivity.

A superacid, trifluoromethanesulfonic acid (TFMS), can lead to enhance the TE properties of the PEDOT:PSS films. Wang et al. demonstrated that the TFMS treatment in methanol (MeOH) on PEDOT:PSS films induced the removal of the insulating PSS and polymer chain rearrangements, giving, in turn, a denser packing of the conductive PEDOT polymer chains [33]. During the dedoping process, TFMS-MeOH as a post-treatment induced the chemical reaction between CF_3_SO_3_H and PSS to generate non-nucleophilic CF_3_SO_3_^−^ anion and the PSSH polyacid, followed by removal of PSSH and the formation of a densely packed crystalline structure. TEMS-MeOH post-treatment increased the electrical conductivity to 2980 S∙cm^−1^ and the Seebeck coefficient to 21.9 µV· K^−1^, translating to a power factor of 142 µW∙m^−1^∙K^−2^.

### 2.3. Poly(3-hexylthiophene) (P3HT)

Poly(3-hexylthiophene) (P3HT) has been widely used in the fabrication of optoelectronic devices due to its excellent electrical properties, appropriate energy gap, and doping reversibility. Easy processability in the solution state makes P3HT suitable for a variety of solution processes such as spray-printing, spin-coating, roll-to-roll printing, and inkjet printing. Zhu et al. investigated the TE performances of P3HT films doped with iodine vapor that were prepared by casting the P3HT solution [34]. Iodine-doped P3HT was conducted by an electron exchange process between iodine as an electron acceptor and P3HT as an electron donor. Upon iodine vapor treatment on pristine P3HT films, the polymer chains self-organized into a more ordered structure, which was confirmed with AFM analysis. They demonstrated that the size of P3HT aggregates was reduced and the number of P3HT single chains increased after doping with iodine vapor, which induced P3HT chains to self-organize into a more ordered structure [34]. As a result, electron transport was enhanced, improving the TE performance. The maximum electrical conductivity of iodine-doped P3HT films was 4.7 × 10^−1^ S∙cm^−1^, which was five orders of magnitude higher relative to that of the counterpart. A comparatively high Seebeck coefficient of 386 µV· K^−1^ was obtained. The calculated power factor was estimated to be 7 µW∙m^−1^∙K^−2^ at room temperature, which was higher than that of the counterpart.

The charge carriers in the conducting polymers transport through the inter-chain and intra-chain hopping processes. Regulating the configuration and arrangement of polymer chains directly affects the carrier transport and, consequently, TE performances. Qu et al. prepared anisotropic P3HT films with a highly oriented morphology using 1,3,5-trichlorobenzene (TCB), an organic small-molecules as a template for polymer epitaxy [35]. In general, P3HT chains adopt an irregular configuration and random arrangement largely due to the flexible hexyl side chains [99], which decreases the carrier mobility and deteriorates the TE performances. However, the resulting P3HT films via a temperature-gradient crystallization process using an organic small-molecule, TCB, as the template for polymer epitaxy exhibited a reduction in π-π conjugation defects along the polymer backbone and also effectively increased the degree of electron delocalization. This produced a large-scale and quasi-1D pathway for the carrier movement and resulted in an enhanced carrier mobility. The electrical conductivity and Seebeck coefficient of the TCB-treated P3HT films were up to 320 S∙cm^−1^ and 269 µV· K^−1^, respectively. As a result, the power factor and ZT value at 365 K reached 62.4 µW∙m^−1^∙K^−2^ and 0.1, respectively, in the direction parallel to the fiber axis.

### 2.4. Polymer/Polymer Thermoelectric Composites

Compounding two or more polymers into one system can be an effective approach for enhancing the TE properties by taking advantages of each polymer. For example, Zhang et al. investigated TE properties of PEDOT nanowire/PEDOT hybrids where PEDOT nanowires synthesized by template-confined in situ polymerization were incorporated into PEDOT:PSS and PDOT:tosylate (Tos), respectively [30]. The TE properties of the hybrids exhibited that the power factor was enhanced by 9 times relative to PEDOT:PSS mixed with 5 vol% DMSO while the low thermal conductivity was maintained. The large enhancement of the TE performance resulted from the synergistic effect of interfacial energy filtering, the nanowire percolation threshold, and the possible changes of carrier concentration in the PEDOT:PSS host materials. Upon the addition of 0.2 wt% PEDOT nanowires to PEDOT:Tos nanocomposites, the power factor of the hybrid materials was increased to 446.6 µW∙m^−1^∙K^−2^ and the ZT reached 0.44 at room temperature.

A novel generation of bilayered nanofilms in which a pure organic PEDOT:PSS nanofilm was obtained via spin-coating techniques and then, followed by depositing P3HT using electrochemical polymerization displayed a good electrochemical stability and enhanced TE performance [36]. Early theoretical simulations indicated that the TE properties of the hybrid composites could not exceed the maximum of each component, but possessed an intermediate between them [37,100]. However, this work by Shi et al. showed that the TE performance could be higher relative to the parent films it was composed of, which could be due to the energy filtering effect created by a potential barrier in nanostructured structures. The electrical conductivity of the resultant PEDOT:PSS/P3HT nanofilms reached up to 200 S∙cm^−1^ along with the Seebeck coefficient of 17 µV∙K^−1^ at 300 K, which translated to a power factor of 5.79 µW∙m^−1^∙K^−2^.

The multilayer structures composed of PEDOT:PSS and PANi-CSA polymers assembled by a layer-by-layer deposition using a spin-coating method enhanced the TE power factor [101]. The multilayer films displayed an enhanced electrical conductivity without sacrificing the Seebeck coefficient, which is typically true in traditional bulk inorganic materials. A PEDOT:PSS/PANi-CSA multilayer exhibited a synergistic improvement in the electrical conductivity, which was likely ascribed to stretching of the backbone chains in both the PEDOT:PSS and PANi-CSA layers. Hole diffusion due to a difference in the Fermi energy between polymers could enhance electrical conductivity. The 4 layers of PEDOT:PSS/PANi-CSA films exhibited a power factor of 56 µW∙m^−1^∙K^−2^.

## 3. Carbon-Based Polymer Nanocomposites

Although the conducting polymers have been shown to be very attractive thermoelectric (TE) materials due to easy processing and environmentally-benign characteristics, along with low thermal conductivity, which is ideal for TE efficiency, their TE properties are still inferior to those of conventional inorganics, primarily due to the low electrical conductivity and Seebeck coefficient [102]. Recently, incorporating carbon nanofillers such as carbon nanotubes (CNTs) and graphene, into the polymer matrix has been proved to be an effective strategy to enhance the electrical conductivity and Seebeck coefficient, resulting in a significant improvement in power factor [17,103]. The unique TE behaviors (i.e., decoupled TE physical parameters) and synergistic enhancement effects have been found in polymer/carbon composites owing to the combination of the advantages of each component and the electrical/thermal transport behaviors at the numerous interfaces [7,102]. In this section, we focus on TE properties of polymers/carbon nanofillers, including graphene and CNTs composites.

### 3.1. Graphene-Based Nanocomposites

#### 3.1.1. PANi-Based Graphene Composites

Wang et al. investigated the TE properties of the HClO_4_-doped PANi/graphite composites, prepared by ball milling and cold pressing, as a function of graphite concentration [38]. Although the thermal conductivity increased with the graphite content, both the electrical conductivity and Seebeck coefficient showed a significant improvement, leading to a large enhancement in the TE performances. When the graphite content increased from 0 to 50 wt%, the electrical conductivity exhibited a dramatic improvement from 1.23 × 10^2^ to 1.2 × 10^4^ S∙cm^−1^ and the Seebeck coefficient also increased from 0.82 to 18.66 µV· K^−1^. The decoupled TE behavior was attributed to the numerous interfaces between the HClO_4_-doped PANi and graphite, which was formed during ball milling. With increasing graphite, there was an obvious improvement in power factor from 8.3 × 10^−5^ to 4.2 µW∙m^−1^∙K^−2^. Therefore, the ZT of the PANi/graphite composites containing a graphite concentration of 50 wt% reached 1.37 × 10^−3^ at 393 K, which was four orders of magnitude higher than the HClO_4_-doped PANi. 

The TE bulk composites pellets and films in which PANi and graphene nanosheets (GNs) were mixed with various ratios displayed an interesting TE behavior [39]. When the weight ratio of PANi to GNs decreased from 4:1 to 1:1, both the electrical conductivity and the Seebeck coefficient simultaneously increased in both pellets and films, which could be explained by a dramatic increase in carrier mobility, while the carrier concentration remained unchanged. The power factor of the pellets was 5.6 µW∙m^−1^∙K^−2^. PANi/GNs nanocomposites, prepared by in situ polymerization of aniline monomer in the presence of GNs, were studied by Xiang et al [104]. They fabricated a paper-like nanocomposite in which PANi grew on the basal plane of GNs via a strong π interaction using controlled vacuum filtration of an aqueous dispersion of PANi. Although GNs are easily agglomerated in the aqueous solution due to the layer structure and the large specific surface area of graphene, the strong π-π interaction between the nuclei of PANi and GNs helped overcome the van der Waals attraction and facilitate uniform dispersion of GNs. GNs served as the template, helped PANi chains more ordered and aligned on the GNs. The aligned PANi chain conformation reduced the carrier hopping resistance, consequently leading to an improvement in carrier mobility and hence Seebeck coefficient. The chemically stretched PANi nanofibril on the surface of GNs as a result of π electron interaction enhanced TE properties, producing a ZT value of 1.5 × 10^−4^ at room temperature.

The dispersion state, the structural defects, and the impurity element content in the graphene can influence the TE properties of the PANi/graphene nanocomposites [40]. PANi/graphene composites, prepared by a solution-assisted dispersing method, showed higher TE properties with graphene having lower structural defects and oxygen impurities. The maximum electrical conductivity and power factor of the composite reached 856 S∙cm^−1^ and 19 µW∙m^−1^∙K^−2^. These outstanding TE properties of PANi/graphene composites were attributed to the strong π-π conjugation interactions between PANi and low-defect graphene. The strong electronic interaction between PANi and graphene induced PANi chains to orient along the surface of graphene with more ordered conformation, which minimized the π-π conjugation defects in PANi molecular chains and resulted in an increase of carrier mobility.

PANi/graphene composites fabricated by a combination of in situ polymerization and a solution process displayed a significant improvement in TE properties [41]. PANi chains were coated on the surface of graphene by the strong π-π conjugation interactions during in situ polymerization, and then the molecular structure of PANi switched coiled conformation to expanded chains by the chemical interactions between PANi and solution. Sequential chemical reactions of in situ polymerization and the solution process created the uniform dispersion of graphene in the PANi matrix and also generated numerous nano-interfaces that could enhance the carrier mobility of the composites. The poor dispersion of graphene due to strong van der Waals interactions between graphene sheets in the polymer matrix usually ends up with a decrease in the Seebeck coefficient, but this work showed that the in situ polymerization process enhanced the dispersion homogeneity of graphene. The enlarged contact surface area provided by uniformly dispersed graphene generated more graphene/PANi interfaces that boosted the energy filtering effect and strengthened the π electronic conjugation structure. The maximum electrical conductivity and Seebeck coefficient of the composite with 48 wt% graphene reached 814 S∙cm^−1^ and 26 µV· K^−1^, respectively, translating to a power factor of 55 µW∙m^−1^∙K^−2^, which is one of the highest values ever reported in the polymer/graphene composites. 

PANi/graphite oxide (GO) prepared via in situ polymerization displayed that the GO played a role of a template where the aniline molecules were grown on the surface of GO, which induced an ordered structure of PANi with a high crystallinity during polymerization [42]. XRD, fourier-transform infrared spectroscopy (FTIR), and XPS confirmed that there existed a strong interaction between exfoliated GO and PANi, including hydrogen bonding, π-π stacking, and electrostatic interaction. The exfoliated GO possessed large surface area, which could render polymers to have strong interactions with GO and hence the PANi chains in the composite were further ordered due to the template effect of GO. PANi/GO exhibited the maximum electrical conductivity and Seebeck coefficient, each of which was up to 750 S∙cm^−1^ and 28 µV· K^−1^, respectively, and the ZT value reached 4.84 × 10^−4^. Mitra et al. investigated the TE performances of the PANi/reduced graphene oxide (rGO) composites by varying its concentration of rGO and PANi [43]. It turned out that the ZT of PANi/rGO increased with an rGO of up to 50%. A uniform growth of PANi with an ordered structure with high crystallinity on the basal plane of the rGO sheets increased carrier mobility, as evidenced by a Hall Effect measurement. The PANi/rGO composites prepared by the in situ chemical oxidative polymerization of aniline exhibited the maximum ZT value of 0.0046, which was 5 times higher than that of pure PANi [105].

#### 3.1.2. Poly(3,4-ethylenedioxythiophene)-poly(styrenesulfonate) (PEDOT:PSS)-Based Graphene Composites

**Figure 4:**
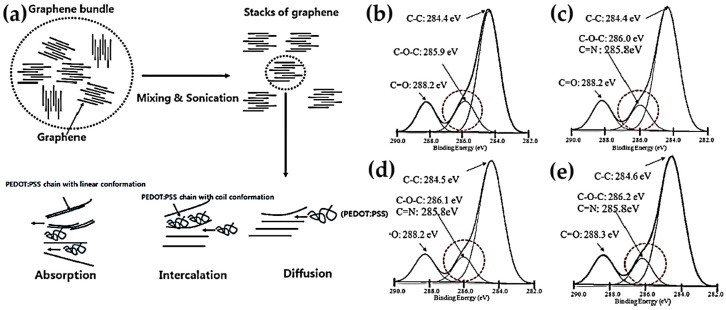
(**a**) The mechanism of dispersion of aggregated graphene in the polymer (PEDOT:PSS) during mixing and sonication processing. C1s x-ray photoelectron spectroscopy (XPS) peaks of (**b**) PEDOT:PSS, (**c**) PEDOT:PSS containing 1 wt% graphene, (**d**) PEDOT:PSS containing 2 wt% graphene, and (**e**) PEDOT:PSS containing 3 wt% graphene. Reprinted with permission from [106]. Copyright 2012 The Royal Society of Chemistry.

PEDOT:PSS graphene composites with a different weight percentage of graphene, prepared by solution spin coating, exhibited an enhancement in TE properties [106]. The stacked graphene was dispersed by shear stresses imposed by the mixing and sonication process, which broke up the aggregated graphene. After dispersing the graphene, the PEDOT:PSS was intercalated into the graphene sheets and there existed strong π-π interactions between each other, as shown in Figure 4a. XPS clearly displayed that the peaks were shifted slightly towards higher binding energy levels, which indicated the electron donation from PEDOT:PSS to the graphene surface and subsequently increased the carrier concentration (Figure 4b). The strong π-π interactions between the PEDOT:PSS and the graphene surface helped to reduce conjugated defects in the polymer backbone because the PEDOT:PSS was tightly coated on the graphene surface. This, in turn, caused the conformational changes of PEDOT:PSS into linear or expanded-coiled molecular structures. The PEDOT:PSS thin films with 2 wt% graphene had the maximum power factor of 11 µW∙m^−1^∙K^−2^ at 300 K and the ZT value was up to 2.1 × 10^−2^.

Doping with bromine can induce phonon scattering by introducing defects and decreasing the thermal conductivity. Ma et al. employed bromine doping into the PEDOT:PSS/graphene fiber to enhance TE performances [44]. Bromine doping made the Fermi level move down to the valence band due to the draining of electrons toward the highly electronegative Br sites. Lowering the Fermi level increased the density of the holes at the band edge, leading to an enhanced electrical conductivity and Seebeck coefficient. With an enhanced transport of charge carriers via bromine doping, the power factor was 624 µW∙m^−1^∙K^−2^ at room temperature. 

PEDOT:PSS/rGO composites where reduced graphene oxide was dispersed in a PSS solution and followed by polymerization with the addition of EDOT monomer to the dispersion exhibited that the TE properties varied with the graphene content [45]. The electrical conductivity increased from 450 to 637 S∙cm^−1^ at 3 wt% of graphene without the need for a reduction step such as the use of a toxic treatment process. Due to the enhanced electrical conductivity, the power factor of the PEDOT:PSS/rGO composites was up to 45.7 µW∙m^−1^∙K^−2^.

Noncovalently functionalized graphene with fullerene by π-π stacking in a liquid-liquid interface could help improve the TE properties of PEDOT:PSS. It was reported that fullerene could reduce the thermal conductivity via phonon scattering [107]. Zhang et al. introduced fullerene into the PEDOT:PSS/graphene composites in which rGO was noncovalently functionalized with fullerene through π-π stacking in the liquid-liquid interface [108]. They found out that tailoring the fullerene and graphene ratio in the PEDOT:PSS matrix could tune the electronic and phonon transport and help increase the electrical conductivity due to the significant interfacial phonon scattering. The incorporation of fullerene/rGO nanohybrids enhanced the Seebeck coefficient as high as 4-fold relative to that of neat PEDOT:PSS film. Employing rGO into the conjugated polymer system could push the Fermi level away from the valence band, resulting in an increased Seebeck coefficient [109]. Zero-dimensional fullerene can allow quantum confinement where high energy carriers preferentially participate with the carrier transport while low energy carriers are impeded [56]. This further enhanced the Seebeck coefficient in the rGO-fullerene/PEDOT:PSS hybrid composites. The highest ZT reached 0.067 at 30 wt% nanohybrids-filled polymer composites, where the ratio of fullerene to graphene was 3:7.

PEDOT:PSS/rGO/fluorinated C_60_ (F-C_60_) hybrid nanocomposites exhibited an enhanced power factor of 83.2 µW∙m^−1^∙K^−2^ [46]. The nanointerfaces created in between rGO, F-C_60_, and PEDOT:PSS provided Schottky barriers, which hindered cold-energy carriers and preferentially allowed high energy carriers to pass through interfacial energy filtering. The synergistic combination of the rGO/F-C_60_ hybrid and PEDOT:PSS formed Schottky barrier due to work functions and resulted in a ZT of 0.1 at room temperature.

#### 3.1.3. Other Conducting Polymers-Based Graphene Composites

Du et al. investigated the TE properties of graphene nanosheets (GN) in the poly(3-hexylthiophene) (P3HT) matrix, which was prepared by oxidative polymerization [47]. As the content of GN increased to 30 wt%, the electrical conductivity was optimized to 1.2 S∙cm^−1^ and the Seebeck coefficient increased to 35.5 µV· K^−1^, resulting in the power factor of the P3HT/GN becoming 0.16 µW∙m^−1^∙K^−2^ at 30 wt% of graphene. A simultaneous increase in the electrical conductivity and Seebeck coefficient was ascribed to an increase in the carrier mobility of the composites with GN loading.

The incorporation of GN into the polypyrrole (PPy) matrix showed an enhanced TE property with a proper ratio of polymer to GN. PPy/GN composites, synthesized by a simple in situ chemical polymerization, exhibited that PPy grew along the surface of GN to form an ordered molecular structure with increased crystallinity [48]. The strong π-π interactions between PPy and GN created during polymerization induced the conformation of PPy molecular chains to change from a compacted coil to an expanded coil. Moreover, the GN was homogeneously dispersed in the PPy matrix and effectively bridged the carrier transport via the π-π interactions with PPy, which, in turn, improved the carrier mobility [110]. By increasing the GN content, the electrical conductivity and Seebeck coefficient enhanced simultaneously. The optimized power factor of the composites reached 10.2 µW∙m^−1^∙K^−2^, which is 250 times higher compared to that of pure PPy [16]. The thermal conductivity was measured to be 0.84 W∙m^−1^∙K^−1^ and therefore, the maximum ZT was up to 2.8 × 10^−3^.

The three-dimensional (3D) interconnected nanocomposite where two-dimensional (2D) rGO was sandwiched by one-dimensional (1D) PPy nanowires has been synthesized via a convenient interfacial adsorption-soft template polymerization [49]. A 3D interconnected microstructure displayed that the PPy nanowires and the rGO nanolayers were connected and formed a 3D network architecture. During polymerization, the PPy nanowires were tightly attached on the surface of the rGO nanolayers, which provided conducting pathways for electron transport. At an rGO:PPy mass ratio of 50 wt%, the nanocomposite reached an electrical conductivity of 75 S∙cm^−1^, which is about 60 times higher than that of the pure PPy nanowires, and the Seebeck coefficient was up to 34 µV· K^−1^, which translated to a power factor of 8.6 µW∙m^−1^∙K^−2^. This value is about 480 times higher relative to that of pure PPy nanowires.

Uniform PPy coating on rGO was fabricated via a template-directed in situ polymerization in which sodium dodecyl sulfate (SDS) was adsorbed on the rGO surface via van der Waals forces and helped disperse rGO [111]. SEM and TEM confirmed that exfoliated rGO flakes acted as the template in the core and PPy wrapped around the rGO as the shell layers. The exfoliated rGO nanosheets with the help of SDS was beneficial for the PPy molecules to have an ordered alignment on the rGO surface, which resulted in a great enhancement in both the electrical conductivity and Seebeck coefficient. The PPy/rGO composites with the rGO:PPy ratio of 2:1 exhibited a power factor of 3 µW∙m^−1^∙K^−2^ at room temperature.

**Figure 5:**
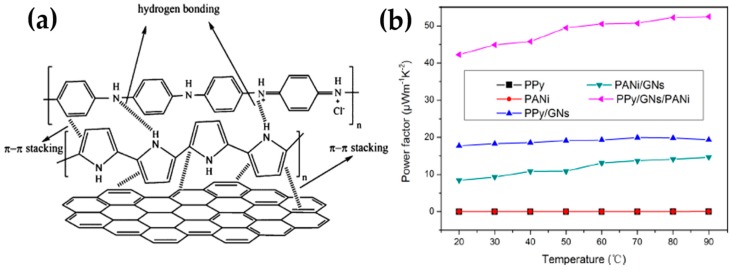
(**a**) The diagram of interactions of polypyrrole (PPy)/graphene nanosheets (GNs)/PANi composite and (**b**) Power factor of pure PPy, PANi, PPy/GNs composite, PANi/GNs composite, and PPy/GNs/PANi composite with 32 wt% graphene at different temperatures. Reprinted with permission from [50]. Copyright 2017 American Chemical Society.

The ternary composites of PPy/GN/PANi synthesized by the combination of the in situ polymerization and solution process exhibited high TE performance, which stemmed from the strong π-π interactions between PPy, GN, and PANi through π-π stacking and hydrogen bonding, as shown in Figure 5a [50]. A uniform coating of PPy on the surface of graphene was achieved from in situ polymerizations, and mixing pure PANi with PPy/GN created the PPy/GN/PANi ternary composites. The highly ordered structure of polymers and the uniform dispersion of graphene in the polymer matrix during polymerization were very beneficial to create numerous interfaces between components, which may cause the increase in the energy filtering and the quantum-confinement effects and consequently enhance the carrier mobility [72]. A highly aligned polymer chain with an ordered molecular structure on the surface of the graphene nanosheets reduced the interchain and intrachain hopping barrier, which significantly lowered the carrier transport resistance. The TE properties were shown to be dramatically improved by combining three components compared to those of two component composites (PANi/GN and PPy/GN), as shown in Figure 5b. At 32 wt% of graphene, PPy/GN/PANi exhibited an electrical conductivity of 500 S∙cm^−1^ and Seebeck coefficient of 32 µV· K^−1^, leading to a maximum power factor of 52.5 µW∙m^−1^∙K^−2^.

### 3.2. Carbon Nanotube-Based Nanocomposites

#### 3.2.1. Non-Conductive Polymers-Based Carbon Nanotubes (CNTs) Composites

Nafion, a water-soluble perfluorosulfonated polymer, has a hydrophobic nature in its backbone that could be used to solubilize CNTs in an aqueous solution. Choi et al. investigated the TE properties of Nafion/CNTs nanocomposites using the doctor blade method with different types of CNTs where single CNTs (SWNT), few CNTs (FWNT), and multi-walled CNTs (MWNT) were dispersed in an aqueous solution of Nafion [51]. For all CNTs studied, both the electrical conductivity and Seebeck coefficient increased with the concentration of CNTs. Although the Seebeck coefficient remained insensitive on the CNTs (ranged to 20–25 µV· K^−1^), the electrical conductivity was dependent on the types of CNTs. Among the CNTs-based composites studied, the Nafion/MWNT films at the 30 wt% of MWNTs exhibited the highest electrical conductivity at 13 S∙cm^−1^. This work indicated that the electrical conductivity was dependent on the types of CNT and that the Seebeck coefficient was relatively insensitive of the CNT type, which indicated that high-energy-charges could participate in transport processes irrespective of the type of CNTs [62]. The maximum power factor was 1 µW∙m^−1^∙K^−2^ in the Nafion/FWNT composites.

Segregated-network polymer/CNT composites investigated by Yu et al. exhibited that the electrical conductivity dramatically increased by creating a network of CNTs in the composite, while the Seebeck coefficient and thermal conductivity were independent upon the CNT concentration [52]. A poly(vinyl acetate) (PVAc) homopolymer emulsion, which is a stable suspension of solids, was used as a matrix for CNTs, and gum arabic (GA) was also used to stabilize the CNT in water. The 3D network of the CNTs was formed within the interstitial spaces between the emulsion particles where CNTs wrapped around the emulsion particles in a network fashion rather than being randomly distributed. The segregated network created thermally disconnected but electrically connected junctions in the nanotube network. With a CNT concentration of 20 wt%, the electrical conductivity and thermal conductivity of the composites were 48 S∙cm^−1^ and 0.34 W∙m^−1^∙K^−1^, respectively, which translated to a ZT of 0.006 at room temperature. Since phonon and electron transport are affected by tube/tube junctions, controlling the carrier transport across the junctions by altering the stabilizer concentration influenced the TE behavior of latex-based polymer composites. Kim et al. investigated the TE behavior of PVAc/SWNT-GA composites by varying the SWNT:GA ratios [53]. The electrical conductivity was significantly increased from 5.27 to 90.4 S∙cm^−1^ by changing the SWNT:GA ratio from 1:3 to 10:1 while the Seebeck coefficient and thermal conductivity showed a little change with varying SWNT:GA ratios, each of which remained 40 µV· K^−1^ and 0.25 W∙m^−1^∙K^−1^, respectively.

**Figure 6:**
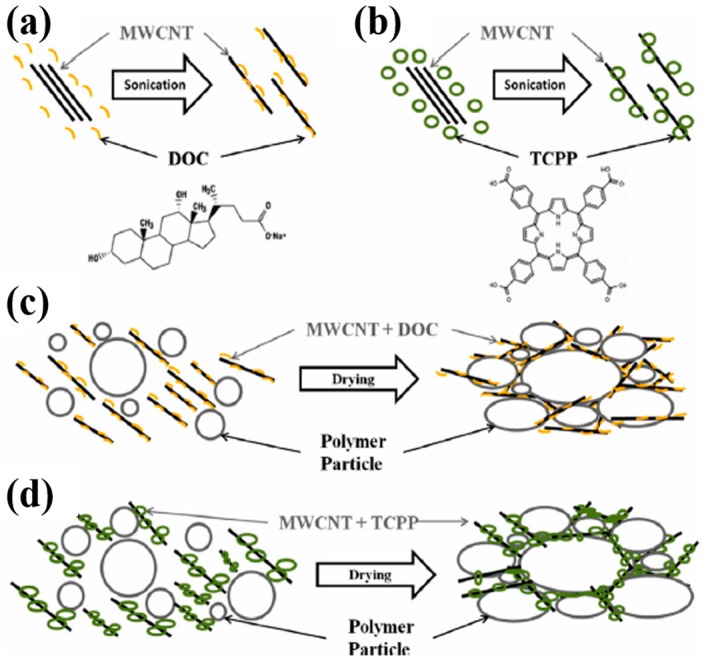
The dispersed multi-walled carbon nanotubes (MWNT), when mixed with polymer emulsion particles, forms a three-dimensional network in the interstitial positions between the polymer particles. (**a**,**b**) show representations of the dispersed MWNT in the two stabilizing agents, DOC and TCPP. (**c**,**d**) illustrate the formation of a segregated network upon the drying of the water-based polymer emulsions. Reprinted with permission from [54]. Copyright 2012 Elsevier.

The same group further investigated the TE behaviors of the CNT-filled latex-based composites, where PVAc was used as a matrix for MWNT, stabilized with a semiconducting molecule, such as sodium deoxycholate (DOC) or meso-tetra(4-carboxyphenyl) (TCPP) [54]. Both surfactants induced the MWNT to be exfoliated in solution by adsorbing to their surfaces and changing them from hydrophobic to hydrophilic. Upon drying, the PAVc polymer chains excluded a volume that the MWNT could occupy, which forced the nanotubes into the interstitial spaces between them and resulted in a segregated MWNT network, as shown in Figure 6. The electrical conductivity of the PVAc/MWNT composites increased at relatively low MWNT concentrations for both stabilizers. The PVAc/MWNT-TCPP composites with 12 wt% MWNT exhibited an electrical conductivity of 1.28 S∙cm^−1^ and a Seebeck coefficient of 28 µV· K^−1^. By replacing MWNT with double walled carbon nanotubes (DWNT), the PVAc/DWNT-TCPP composites revealed an electrical conductivity of 71.08 S∙cm^−1^ and Seebeck coefficient of 78 µV· K^−1^, which translated to a power factor of 42.8 µW∙m^−1^∙K^−2^.

#### 3.2.2. PANi-Based CNTs Composites

Meng et al. demonstrated a novel approach to enhancing TE properties of the PANi/CNT nanocomposites prepared via a simple two-step method [56]. A freestanding CNT network consisted of individual CNTs and their bundles were fabricated by filtering a uniform CNT suspension. A PANi layer was coated on the CNT network via the in situ chemical polymerization method. By incorporating the CNT (1D materials) and PANi (quasi-1D materials) that exhibit larger TE properties than 2D or 3D ones due to a higher density of states at the Fermi level in low-dimensional structures, the resultant PANi/CNT composites enhanced the power factor to 5 µW∙m^−1^∙K^−2^ at 300 K [55,112]. PANi/SWNT nanocomposites prepared via a simple one-step in situ polymerization by using SWNTs as templates and aniline as reactant displayed the nanostructured PANi coating layer tightly wrapped around the SWNTs [60]. With increasing SWNT content, both the electrical conductivity and Seebeck coefficient of PANi/SWNT were enhanced. When the SWNT content was varied from 0 to 41.4 wt%, the electrical conductivity reached 125 S∙cm^−1^ and the Seebeck coefficient increased from 11 to 40 µV· K^−1^. Such a dramatic improvement in the TE properties resulted from the increase of the carrier mobility in PANi that were aligned along the SWNTs with a good order of chain packing. The SWNTs further increased the carrier mobility by bridging the carrier transport through the strong π-π interactions with the rings of PANi. The maximum power factor at room temperature was up to 20 µW∙m^−1^∙K^−2^ for the 41.4 wt% SWNT and the thermal conductivity was 1.5 W∙m^−1^∙K^−1^, which translated to a maximum ZT of 0.004 at room temperature.

The PANi/CNT composites with an ordered molecular structure were also obtained by a combination of in situ polymerization and electron-spinning processes [58]. During in situ polymerization, aniline molecules were polymerized and aligned along the CNTs with a high degree of ordering due to the strong PANi and CNT interaction. Furthermore, the electrical field in the electro-spinning process caused PANi/CNT hybrids to align in a parallel manner into a long fiber. The highly ordered arrangement of polymer chains reduced the π-π conjugated defects and increased the effective degree of electron delocalization, which enhanced the carrier mobility in the composites [113]. The PANi/CNT nanofibers exhibited an electrical conductivity of 17 S∙cm^−1^ and Seebeck coefficient of 10 µV· K^−1^ with CNT of 40 wt%. Zhang et al. prepared PANi/MWNT nanocomposites in a green method with no use of a dispersant [57]; that is, PANi and MWNTs were homogeneously mixed by cryogenic grinding (CG) and then the PANi/MWNT composites were consolidated by Spark Plasma Sintering (SPS). Upon SPS treatment, the electrical conductivity of the PANi/MWNT nanocomposites increased to 1.6 S∙cm^−1^ by increasing the MWNT content from 10 to 30%, and the maximum power factor was 1.1 µW∙m^−1^∙K^−2^. 

The in situ electrochemical polymerization process makes it possible for the polymer composites to adjust the morphology and microstructure. Liu et al. fabricated flexible PANi/SWNT composites and investigated the effect of the microstructure on their TE performance by modulating the electrolyte component and electric current [59]. During the electro-polymerization process, the conjugated structure of PANi chains was deposited onto the π-bonded surface of the SWNTs. PANi worked as the conductive glue to assemble the SWNTs into a homogeneous conductive network which facilitated the transfer of charge carriers. The SWNT network acted as a self-assembly template and accelerated the nucleation and the growth of PANi. SEM and TEM confirmed that more PANi was progressively electrodeposited onto SWNT with the increase in the diameter of PANi/SWNT composites. While the electro-polymerization proceeded, the composite films went through structural transitions to form 3-dimensional networks. The PANi/SWNT composites exhibited an electrical conductivity of 32 S∙cm^−1^ and Seebeck coefficient of 45 µV· K^−1^ when the number of electro-polymerization reached 75. Further electrochemical polymerization made disordered PANi deposited, which resulted in the decrease in TE properties. The calculated power factor was up to 6.5 µW∙m^−1^∙K^−2^.

**Figure 7:**
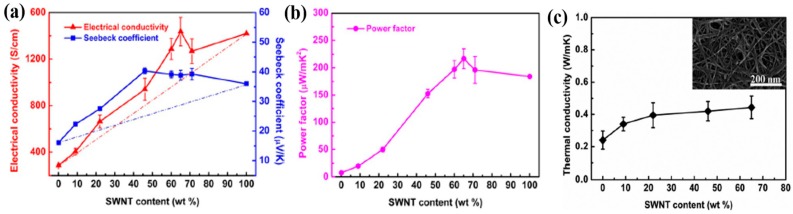
(**a**) The electrical conductivity and Seebeck coefficient, (**b**) power factor, and (**c**) thermal conductivity at room temperature of the PANi/single carbon nanotubes (SWNT) composites. The inset is the SEM image of PANi/SWNT composites with SWNT content of 65 wt%. Reprinted with permission from [61]. Copyright 2016 John Wiley and Sons.

Regulating the degree of ordering of the molecular chain arrangements is an effective way to increase the electrical transport properties in organic TE materials. Wang et al. prepared PANi/SWNT nanocomposites by combining in situ polymerization and m-cresol solution processing [61]. During in situ polymerization SWNTs were well-dispersed in the nanocomposite and formed strong π-π interactions with PANi. The PANi/SWNT composite with SWNTs of 65 wt% exhibited an electrical conductivity of 1440 S∙cm^−1^ and Seebeck coefficient of 39 µV· K^−1^, which translated to a power factor of 217 µW∙m^−1^∙K^−2^ at room temperature. This value was more than 20 times higher relative to that of the pure PANi film (Figure 7a,b). A large enhancement of TE properties in the nanocomposites was attributed to the highly ordered structure of PANi chains along the SWNTs via strong π-π interactions, which decreased the interchain and intrachain defects of the PANi molecules and consequently increased the carrier mobility. The Hall Effect measurement indicated that the carrier concentration slightly increased while the carrier mobility tripled with the SWNT contents. This result revealed that a simultaneous enhancement in both the electrical conductivity and Seebeck coefficient was mainly ascribed to the increase in carrier mobility. The thermal conductivity, measured by the laser flash technique at room temperature in the out-of-plane direction, was in the range of 0.2 to 0.5 W∙m^−1^∙K^−1^ with different SWNT contents, as shown in Figure 7c. This low thermal conductivity could be explained by the fact that the numerous nano-interfaces formed in between PANi and SWNTs effectively scattered phonons and the differences in the vibrational spectra hindered phonon transport in the composites [114].

#### 3.2.3. PEDOT:PSS-Based CNTs Composites

The decoupled TE behaviors, which are ideal for realizing practical TE devices, between physical parameters such as electrical conductivity, Seebeck coefficient, and thermal conductivity can be achieved by modifying junctions between polymers and CNTs. A segregated network polymer-CNT composite created by drying water-based polymer emulsions that occurred after the addition of CNTs, stabilized in PEDOT:PSS, exhibited electrically connected, but thermally disconnected junctions [62]. With a high SWNT concentration of 35 wt%, the electrical conductivity was measured to be 400 S∙cm^−1^, but the Seebeck coefficient was relatively insensitive to the change, ranging from 15 to 30 µV· K^−1^. The maximum power factor was up to 25 µW∙m^−1^∙K^−2^. The large improvement in electrical conductivity by raising SWNT loading did not affect the thermal conductivity of the DMSO doped-PEDOT:PSS/SWNT composites, which remained more or less the same in the range of 0.2–0.4 W∙m^−1^∙K^−1^. The TE properties of the segregated polymer-CNT network structure were enhanced by combining PEDOT:PSS, polyvinyl acetate, and SWNT [63]. Yu et al. investigated the TE behaviors of the polymer/SWNT composites consisting of SWNTs mixed with different grade PEDOT:PSS and/or PVAc polymers. Among samples investigated in their study, PEDOT:PSS (PH1000 type, 30 wt%)/PVAc (10 wt%)/SWNT (60 wt%) composites exhibited the highest TE performance with a power factor of 160 µW∙m^−1^∙K^−2^.

**Figure 8:**
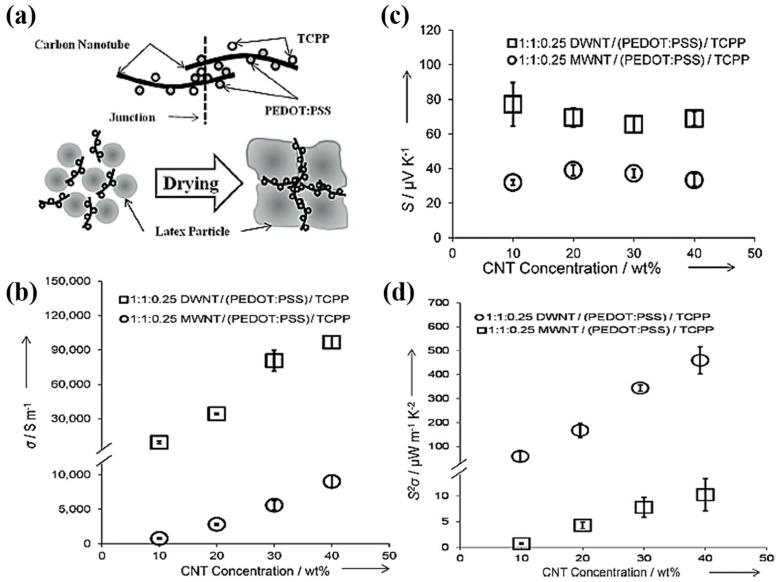
(**a**) top: the schematic of double walled carbon nanotubes (DWNT) coated by PEDOT:PSS and TCPP molecules and the junction formed between them. Bottom: schematic of the formation of a segregated network composite during polymer coalescence as it dries, (**b**) electrical conductivity, (**c**) seebeck coefficient, and (**d**) power factor as a function of CNT concentration and type. Reprinted with permission from [64]. Copyright 2013 John Wiley and Sons.

The same research group further improved the TE performance by using a dual-dispersants of TCC and PEDOT:PSS, which are intrinsically conductive and semiconducting stabilizers, respectively, for polymer/CNT composites [64]. In this work, two types of CNTs, including MWNT and DWNT, were exfoliated by using the combination of TCPP and PEDOT:PSS, both of which have been shown to stabilize CNTs through strong π-π interactions. Segregated-network polymer composites were created by embedding uniformly dispersed CNTs in TCPP and PEDOT:PSS into PVAc latex and followed by drying water-based polymer emulsions at an elevated temperature. As the water evaporated during drying, the polymer particles pushed the CNTs into interstitial spaces to form an electrically connected, but thermally disconnected 3D network. A composite made with PVAc latex and 40 wt% MWNTs 1:1:0.25 MWNT/PEDOT:PSS/TCPP exhibited 95 S∙cm^−1^ (Figure 8a). The high electrical conductivity could be attributed to more favorable junctions between multiple stabilizing agents and CNTs in the segregated-network. An electrical conductivity of the sample system after replacing MWNTs with DWNTs was significantly improved by one order of magnitude (960 S∙cm^−1^) and the Seebeck coefficient was nearly doubled to 70 µV· K^−1^, which translated to a power factor of 500 µW∙m^−1^∙K^−2^ in the PVAc/PEDOT:PSS/TCPP/DWNT composites (Figure 8b–d).

Zhang et al. reported that the TE properties of PEDOT:PSS/MWNT nanocomposites were greatly enhanced by a template-directed in situ polymerization [115]. Monomeric 3,4-ethylenedioxythiophene was adsorbed onto the MWNT that had been stabilized in PSS and polymerized after adding ammonium peroxydisulfate and iron chloride. Due to the strong π-π interaction and van der Waals interactions, PEDOT:PSS layers were effectively formed and coated on the surface of the MWNTs. At a ratio of PSS:EDOT = 0.3:1 and an MWNT:EDOT mass ratio of 70 wt%, the power factor of the PEDOT:PSS/MWNT nanocomposites reached a value of 0.23 µW∙m^−1^∙K^−2^.

The PEDOT:PSS/SWNT composites with a layered nanostructure were fabricated via two-step spin casting [65]. The double-layer nanostructure of the PEDOT:PSS/SWNT composite displayed a simultaneous improvement in electrical conductivity and Seebeck coefficient without the addition of dielectric solvents. The decoupled TE behavior was ascribed to the energy filtering effect. The layered nanostructure of PEDOT:PSS and SWNTs formed numerous nanometer-sized interfaces between each component that could create energy potential barriers. The maximum electrical conductivity and Seebeck coefficient of the PEDOT:PSS/SWNT composites reached 240 S∙cm^−1^ and 39 µV· K^−1^, respectively. The power factor was up to 21 µW∙m^−1^∙K^−2^, which is 4 orders of magnitude higher than the pure PEDOT:PSS.

Ethylene glycol (EG) treatment is an effective way to improve the electronic property of PEDOT:PSS-based composites by selectively removing non-conductive PSS chains out of the PEDOT:PSS films [31,116]. Lee et al. investigated the post-treatment of PEDOT:PSS/CNT nanocomposites by simply immersing the as-prepared films into the EG for 1 h, followed by annealing at 140 °C for 10 min to remove the residual EG [66]. Prior to post-treatment, the nanocomposite films with 25% DWNT exhibited a relatively low power factor of 38 µW∙m^−1^∙K^−2^. However, when subjected to EG treatment, the power factor of the nanocomposites dramatically increased up to 150 µW∙m^−1^∙K^−2^. The removal of the non-complexed PSS chains decreased the inter-CNT bundle distance and reduced the tunneling distance, consequently increasing the total carrier density of states and, hence, the electrical conductivity of the nanocomposite films.

#### 3.2.4. Other Conducting Polymers-Based CNTs Composites

Other conducting polymers have been shown to be effective TE materials with high performances when compounded with carbon nanotubes. P3HT/SWNT nanocomposite films prepared by using simple-bar-coating exhibited a high TE performance without additional P3HT doping. Lee et al. investigated the TE properties of P3HT/SWNT films by optimizing the SWNT composition and solid content in the wire-bar-coating process [68]. Compared to drop-cast films, the wire-bar-coated P3HT/SWNT nanocomposite films, where SWNTs with diameters in the range of 6–23 nm formed well-dispersed and interconnected networks, exhibited a much higher TE performance with power factor up to 105 µW∙m^−1^∙K^−2^ at room temperature for an ink with a total solid content of 4 mg mL^−1^. Combining P3HT with MWNT via solution mixing showed a relatively high TE performance due to a uniformly dispersed MWNT network in the P3HT matrix [67]. The electrical conductivity of P3HT/MWNT increased remarkably from 0.02 to 30 S∙cm^−1^ with an MWNT content from 10 to 90 wt%, and the Seebeck coefficient remained constant to the values between 23 and 28 µV· K^−1^, while the thermal conductivity slightly increased to 0.59 W∙m^−1^∙K^−1^. More conductive pathways through the composites with increasing MWNT content improved the ZT value to 8.71 × 10^−4^ at 80 wt% of MWNT.

Bounioux et al. reported a high TE performance of the P3HT/SWNT composites that were compounded with SWNT followed by optimized p-doping [69]. Optimally p-doped P3HT/SWNT composites revealed an electrical conductivity of 1000 S∙cm^−1^ and produced a power factor of 107 µW∙m^−1^∙K^−2^ for 80 wt% SWNT. The TE properties can be dramatically enhanced by the sufficient doping of polymers in the hybrid films. Hong et al. doped P3HT chains with a 0.03 M FeCl_3_/nitromethane solution using simple spin-coating onto the as-prepared P3HT/SWNT hybrid films [70]. During the doping process, dopants diffused into the polymer chains and captured electrons from them, which increased the electrical conductivity of the polymers while SWNT remained insensitive. The resultant P3HT/SWNT composites prepared by a simple bar-coating process exhibited an electrical conductivity of 2760 S∙cm^−1^ and Seebeck coefficient of 31 µV· K^−1^, producing a power factor of 308 µW∙m^−1^∙K^−2^, which tripled that of the hybrid films via conventional immersion method. Spray-printed P3HT/CNT nanocomposites exhibited an excellent TE performance [71]. The electrical conductivity and Seebeck coefficient were up to 348 S∙cm^−1^ and 97 µV· K^−1^, respectively, producing a power factor of 325 µW∙m^−1^∙K^−2^ at room temperature.

Wang et al. fabricated PPy/MWNT composite powders by varying MWNT contents ranging from 0 to 20 wt% via an in-situ polymerization method using p-toluenesulfonic acid as a dopant and iron chloride as an oxidant [72]. The PPy/MWNT nanocomposites, composed of PPy nanoparticles and an MWNT core/PPY shell, exhibited that the electrical conductivity increased to 72 S∙cm^−1^ with an MWNT of 15 wt%, which was more than 3 times as high as that of the pure PPy. An increase in TE properties was attributed to a conductive network where PPy chains were aligned onto MWNT with a more ordered crystalline structure via the π-π interaction between PPy and MWNT, which enhanced the carrier mobility. The PPy/MWNT nanocomposites at 20 wt% MWNT revealed a power factor of 2 µW∙m^−1^∙K^−2^ at room temperature, which was about 26 times higher relative than that of pure PPy.

The free-standing PPy/SWNT composite films, where nanosheets of SWNT, stabilized in SDBS, were physically mixed with PPy nanowires using the convenient procedure of solution mixing and subsequent common vacuum filtration, displayed a unique morphology with a high TE performance [73]. The pure PPy nanowires, synthesized by chemical oxidative polymerization using cetyltrimethyl bromide (CTAB) and ammonium peroxydisulfate (APS), exhibited an electrical conductivity of 2.2 S∙cm^−1^ and a Seebeck coefficient of 10 µV· K^−1^, producing a power factor as low as 0.02 µW∙m^−1^∙K^−2^. PPy/SWNT composites with a layered morphology in which parallel SWNT nanosheets were sandwiched by PPy nanowires revealed an enhanced TE performance with the power factor as large as 21.7 µW∙m^−1^∙K^−2^, which was about 1000 times higher than that of the neat PPy nanowires.

### 3.3. Graphene/CNT-Based Nanocomposites

Hybridization by incorporating 1D and 2D carbonaceous nanofillers into a conducting polymer matrix has been shown to be an effective route for further improving TE properties via the synergistic effects from each component. Employing both low-dimensional carbon materials such as carbon nanotubes and graphene sheets into polymers has exhibited a dramatic enhancement in TE performance. For example, Yoo et al. fabricated a composite hybridized with graphene sheets and MWNT via the in situ polymerization of PEDOT:PSS in an aqueous solution [74]. The MWNT with a high aspect ratio (>1000) and graphene with a large surface area would provide long-range electrical bridges between conductive domains, which lowered the electrical transport resistance. The hybrid composite of PEDOT:PSS/graphene/MWNT with 5 wt% carbon materials exhibited an electrical conductivity and Seebeck coefficient of 690 S∙cm^−1^ and 23 µV· K^−1^, respectively, which resulted in a power factor of 37 µW∙m^−1^∙K^−2^. Along with the thermal conductivity of 0.36 W∙m^−1^∙K^−1^, the ZT of the PEDOT:PSS/graphene/MWNT composite reached 0.31 at room temperature. The enhanced TE performance originated from the synergistic effects of multi-component systems with an excellent electrical bridging and electronic coupling between conductive domains.

Li et al. investigated the TE behaviors of polymer-based ternary composites which were synthesized by the in situ chemical oxidation of EDOT monomers on rGO and followed by physical mixing with SWNT [75]. Strong π-π interactions made the exfoliated SWNT adsorbed on the surfaces of the PEDOT/rGO composites. The resultant ternary composites of PEDOT/rGO/SWNT showed that the conducting networks consisting of 2D dispersed rGO nanosheets (surrounded with PEDOT polymers) and de-bundled SWNTs. Although the pure PEDOT had an electrical conductivity as low as 7.1 S∙cm^−1^, the electrical conductivity of the PEDOT/rGO/SWNT composites was up to 38 S∙cm^−1^. The post-treatment by H_2_SO_4_ further enhanced the electrical conductivity up to as high as 208 S∙cm^−1^. With a slight decrease in Seebeck coefficient after the post-treatment, the ternary composites displayed increases in the power factors with increasing SWNT contents. The H_2_SO_4_-treated PEDOT/rGO/SWNT composites at 10% SWNT revealed a power factor of 9 µW∙m^−1^∙K^−2^.

**Figure 9:**
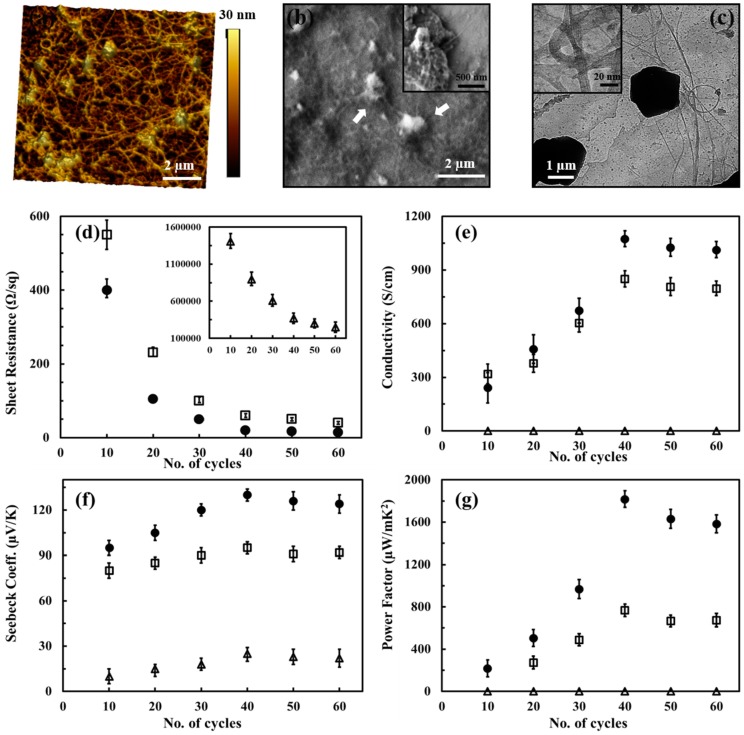
(**a**) The AFM 3D height image, (**b**) SEM, and (**c**) TEM images of the thin films. The insets in the SEM and TEM images show higher resolution graphene platelets and DWNT, respectively. The arrows indicate graphene in the film. (**d**) Sheet resistance, (**e**) electrical conductivity, (**f**) seebeck coefficient, and (**g**) power factor of PANi/graphene (open triangles), PANi/DWNT (open squares), and PANi/graphene/PANi/DWNT (filled circles) as a function of bilayers or quadlayers (i.e., cycles) deposited on a poly(ethylene terephthalate) (PET) substrate. Reprinted with permission from [76]. Copyright 2015 John Wiley and Sons.

The hybridization of carbonaceous nanofillers and conducting polymers by using layer-by-layer (LbL) was reported as a unique way to fabricate nanostructured composites with great potential to improve the TE performances [81,117]. A key advantage of the LbL technique is that of the sequential assembly of intrinsically conductive polymer and carbonaceous nanoparticle networks that leads to a novel three-dimensional film whose TE properties exceed those of each individual component, as well as those of a bulk film made with the same components. For instance, Cho et al. fabricated PANi-based carbon nanocomposites via LbL assembly where DWNT and graphene were stabilized with sodium dodecylbenzenesulfonate (SDBS) and poly(4-stryrenesulfonic acid) (PSS), respectively [76]. AFM on the surface of the PANi/graphene/PANi/DWNT thin films displayed that DWNTs were interwoven with each other, creating nanotube bundles due to their high concentration (Figure 9a). The addition of surfactants allowed DWNT and graphene to be exfoliated and uniformly dispersed in the LbL thin films, forming a 3D network with polymer-like entanglements of nanotubes and graphene platelets. SEM images in Figure 9b showed that individual DWNT and their bundles randomly intertwined together to form a well-dispersed network. TEM in Figure 9c exhibited that two overlapped graphene sheets (average diameter 1–1.5 μm) are surrounded by a PANi–DWNT matrix, where the PANi and DWNT were an interconnected network. Ordered PANi/graphene/PANi/DWNT nanocomposites with a uniform alignment of the 3D network structure yielded a power factor of 1825 µW∙m^−1^∙K^−2^. By fully taking advantage of the nanoscale engineering using the LbL approach, the PANi, graphene, and DWNT in the QL films exhibited a strong synergistic effect that surpassed the bilayer (BL)-LbL nanocomposites (PANi/graphene and PANi/DWNT), without sacrificing the intrinsic electrical properties of the individual carbon components (Figure 9d–g).

By replacing all insulating stabilizers (PSS and SDBS) with water-soluble (or dispersible) intrinsically conductive polymers, PEDOT:PSS, an 80 quadlayer thin film (≈1 μm thick), comprised of a PANi/graphene-PEDOT:PSS/PANi/DWNT-PEDOT:PSS repeating sequence, exhibited an unprecedented electrical conductivity of 1.9 × 10^3^ S∙cm^−1^ and a Seebeck coefficient of 120 μV∙K^−1^ [77] These two values yielded a power factor of 2710 µW∙m^−1^∙K^−2^.

### 3.4. N-Type Thermoelectric Nanocomposites

In order to achieve practical applications, it is simultaneously required to fabricate both efficient p-type and n-type materials with a high TE performance. Recently significant progress in p-type materials has resulted in a high TE performance, however, most n-type counterparts have lower TE properties owing, in a large part, to difficulties in stable doping of organic materials and the lack of efficient n-type doped materials [81,118]. The large differences in the power factor between p- and n-type materials limit the widespread use of organic TE devices because of the unbalanced transport coefficients, which leads to power losses in π-leg module structures [119]. Semiconducting nanotubes are intrinsically of the n-type, but converted into p-type in the air due to their susceptibility on oxygen doping [120]. That is, n-type semiconducting organic materials such as carbon nanotubes (CNTs) are susceptible to oxygen doping in the air and the Seebeck coefficient becomes positive over time due to absorbed O_2_ molecules that withdraw ~1/10 of an electron from CNTs [120,121,122]. To utilize the full potential of TE devices, it is critical to developing n-type polymers and composites to pair with their p-type counterparts. Towards addressing a lack of high-performance n-type characteristics of organic materials, great efforts have been paid to develop new strategies to fabricate n-type TE materials having excellent properties along with a high stability in the air [123,124,125].

All-polymer films have been attempted to produce air-stable n-type TE materials. For example, Sun et al. reported powder-pressed n-type TE films, assembled with conducting polymers poly(K_x_(Ni-1,1,2,2-ethenetetrathiolate)s) (poly(Ni-ett)s), achieving a stable high power factor of 66 µW∙m^−1^∙K^−2^ and a ZT value of 0.2 [78]. However, these polymers are neither soluble nor fusible, which significantly limits their processability. CNTs have been shown to be effectively converted into n-type TE performances through various methods. Solution-processed air-stable n-type TE materials were developed by synthesizing cobaltocene-encapsulated SWNTs [79]. The SWNT film doped with cobaltocene as an n-type showed a large power factor (75.4 μW·m^−1^·K^−2^) and low thermal conductivity of 0.15 W∙m^−1^∙K^−1^, reaching a ZT value of 0.157 at 320 K. Nonoguchi et al. demonstrated stable and efficient n-type SWNT doping with a series of salts such as sodium chloride, sodium hydroxide, and potassium hydroxide with crown ethers [126]. Their new n-type TE materials exhibited a ZT of 0.1 with an unprecedented air stability even at 100 °C for more than 30 days.

A high TE property of n-type CNTs-based composites has been obtained by decorating them with organic such as polyethylenimine (PEI) [127]. The donation of electrons from PEI to DWNTs effectively converted p-type nanotubes into n-type PEI/DWNT composites. After decorating DWNT with PEI through a stirring process, the Seebeck coefficient became −58 μV∙K^−1^. Freeman et al. improved an n-type behavior of CNTs-filled PEI composites [128]. They dispersed CNTs in SDBS under a tip sonication process and added 5 wt% of PEI solution into CNTs solutions in which PEI was expected to be attached on the surface of CNTs by physisorption. The PEI-coated CNTs were then made into composites with polyvinyl acetate (PVAc). The resultant composites exhibited an electrical conductivity of 15 S∙cm^−1^ and Seebeck coefficient as large as −100 μV∙K^−1^. A high n-type behavior was believed to be due to the increase in the number of tubes that were evenly coated with PEI in well-dispersed CNTs in SDBS. The physical adsorption of PEI on the nanotubes made CNTs electron-rich due to the electron transfer from the amine groups in PEI. The lone pairs of the amine groups in PEI effectively donated electrons into the CNTs, resulting in an upward shift of the Fermi energy compared to an initial energy state and thus converting p-type CNTs into n-type. The n-type TE behaviors based on CNTs and PEI were further enhanced by the same group [80]. After functionalizing the CNTs with PEI, sodium borohydride (NaBH_4_) was used as an n-type doping agent for CNTs. NaBH_4_ could dope the CNTs where there were left undoped by PEI, which further improved the n-type characteristics. Therefore, the combination of PEI and NaBH_4_ doping resulted in more effective n-type doping relative to the PEI-doped counterparts. The electrical conductivity of the composites was as high as 240 S∙cm^−1^ and the Seebeck coefficient was −80 μV∙K^−1^. 

**Figure 10:**
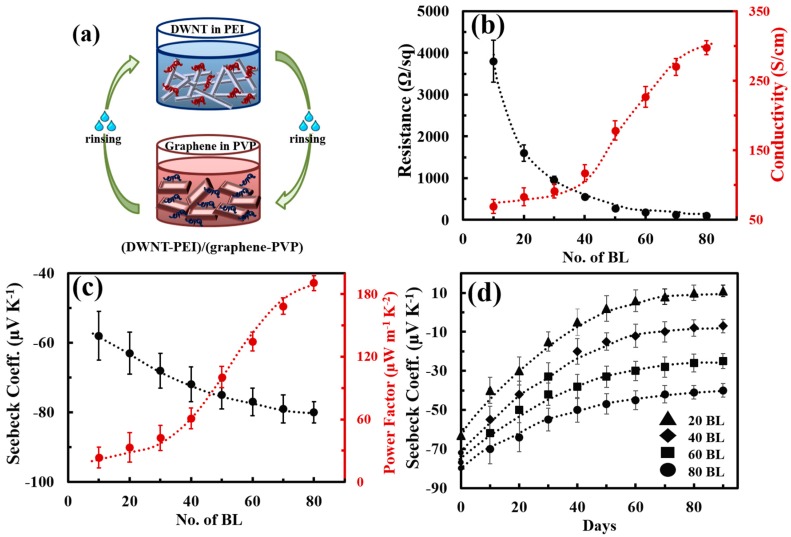
(**a**) The schematic of the layer-by-layer deposition process and molecular structures of the materials used. (**b**) Sheet resistance and electrical conductivity and (**c**) Seebeck coefficient and power factor, and (**d**) air-stability of Seebeck coefficient under ambient conditions. Reprinted with permission from [81]. Copyright 2016 Elsevier.

A high power factor of graphene and carbon nanotubes polymer composites was achieved by using an LbL method in which DWNT-PEI/graphene-PVP were fabricated by alternately depositing DWNT, stabilized by PEI, and graphene stabilized by polyvinylpyrrolidone (PVP), from water, as shown in Figure 10a [81]. The electrical conductivity increased in an absolute form with the number of layers deposited, which suggests an increase in the density of the intersecting pathways for electron transport as the conductive network formed by the nanotubes; the graphene transitioned from 2D to 3D with the increasing layers (Figure 10b). The nanocomposites exhibited a modest increase in the Seebeck coefficient with thickness, attaining 80 µV· K^−1^ (Figure 10c). Both PEI and PVP contain nitrogen atoms either in the backbone or in the side group of the polymer chains, which allows them to act as electron donors. An 80-bilayer DWNT-PEI/graphene-PVP thin film (~320 nm in thickness) revealed a power factor of 190 µW∙m^−1^∙K^−2^ at room temperature. Furthermore, unlike most organic n-type materials, this unique nanocomposite was relatively air-stable, which was demonstrated by testing after 60 days with no protection against moisture and oxygen (Figure 10d). An air-stable TE thin film assembly was believed to be due to the fact that LbL deposition produced nanocomposites of highly aligned and exfoliated graphene layers that created an extreme tortuosity for gas diffusion.

The addition of polyethylene glycol (PEG) into SWNT-based polymer composites is an effective way to convert p-type to n-type TE materials. Luo et al. prepared polypropylene (PP)/SWNT composites through a melting process [82]. By increasing the SWNT content from 0.8 to 2.0 wt%, the electrical conductivity of the PP/SWNT composites increased. The addition of a p-type nanoparticle, CuO, improved the electrical conductivity due to more charge carriers injected into composites. Interestingly, upon the addition of 10 wt% PEG into 2 wt% SWNTs and 5 wt% CuO composites, the Seebeck coefficient was changed from 37 to −57 µV· K^−1^_,_ and the power factor of the PP/SWNT/CuO/PEG composites increased to 7.8 × 10^−2^ µW∙m^−1^∙K^−2^. 

A dramatic improvement in the power factor of the n-type organic materials has been recently made by judiciously controlling the electronic structure of spun carbon nanotube webs using various molecular dopants [83]. An et al. investigated the TE behaviors of a CNT web through the doping characteristics of n-type dopants and annealing treatment to remove oxygen from the surfaces of CNTs. To further improve the n-type TE performance, a benzyl viologen (BV) molecular dopant with the lowest reduction potentials among n-type organic molecules was used instead of an amine-rich polymer, PEI. The use of BV made the Fermi energy level of the CNT web further shift upward to the conduction band. Annealing the p-type CNT web at 300 °C for 10 h, followed by immersion into 2 mg mL^−1^ of BV solution for 8 h, increased the electrical conductivity to as high as 2220 S∙cm^−1^ and the Seebeck coefficient up to −116 µV· K^−1^. The annealed CNT web with BV treatment exhibited a power factor of 3100 µW∙m^−1^∙K^−2^, which is one of the highest values ever reported in organic composites.

## 4. Flexible Organic Modules

Thermoelectric generators (TEGs) have been recognized as a useful waste heat recovery system due to their simple structure, high power density, and lack of noise pollution. Typically, TEGs consist of legs of alternating p-type and n-type materials, each of which is connected electrically in series and thermally in parallel. The maximum heat to electrical energy conversion efficiency (η_max_) of a TE material depends on its Carnot efficiency and TE properties. In TEGs, the efficiency of η_max_ is expressed as [129]

ηmax=Th−TcTh 1+ZTm−11+ZTm+TcTh

where T_m_ = (T_h_ + T_c_)/2, and T_c_ and T_h_ are the cold-side and hot-side temperatures of the device, respectively. Although traditional inorganic-based bulk TE materials have achieved ZT values over 2 along with high power output and power density, this high value is measured at temperatures above 600 K. The ZT of these materials drops below 0.5 with a power factor <400 µW∙m^−1^∙K^−2^ at room temperature [130]. In other words, most inorganic TE materials generate a greater amount of electricity at elevated temperatures (>500 K) [129]. This has restricted their power generation applications for energy harvesting from a variety of heat sources [15]. Considering that more than 50% of waste heat is stored at temperatures <500 K, the use of organic TE materials for the efficient harvesting of waste heat in the 300–500 K range could offer versatile power generation by fully capturing low-graded heat [131]. It is apparent that there will be a growing demand to develop flexible TEGs that could be attached to the device or on curved surfaces like human bodies with recent advances in flexible and stretchable electronic devices. For example, harvesting the heat dissipated from the human body could charge the batteries of small electronics or run low-power devices including medical sensors and wristwatches simply by embedding TEGs into an article of clothing. In this regard, the organic TE nanocomposites characterized by mechanical flexibility and the ready availability of such heat sources in daily life are suitable for developing wearable power generators. The proper utilization of the abundant low-grade heat waste through organic TEGs is a promising technology for various niche applications.

**Figure 11:**
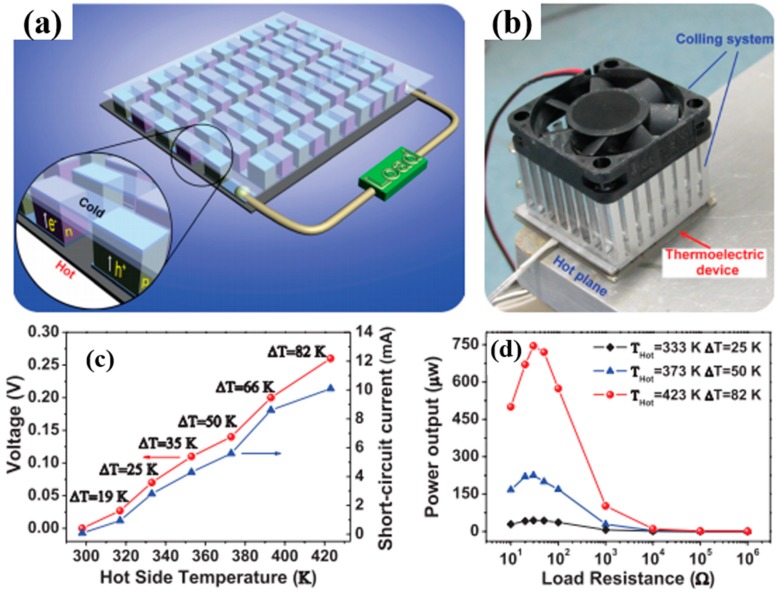
A thermoelectric module consisting of 35 thermocouples. (**a**) Module structure. (**b**) Photograph of the module and the measurement system with a hot plane and cooling fan. (**c**) The output voltage and short-circuit current at various Thot and ΔT. (**d**) The measured power output of the module with different loads. Reprinted with permission from [78]. Copyright 2012 John Wiley and Sons.

All polymer-based p- and n-type TE devices showed high power output performance. Sun et al. fabricated an inkjet-printed flexible device (composed of p- and n-type composites) that was obtained by ball-milling the poly[Ax(M-ett)] (A = Na, K; M = Ni, Cu) with a poly(vinylidene fluoride) solution. An all-polymer TE module consisting of six thermocouples that were printed onto the PET substrate produced a maximum output voltage of 15 mV and a maximum output power of 45 nW with a load resistance of 5 KΩ upon the application of a 25 K temperature gradient. The same research group developed high-performance organic TEGs by combining n-type poly[Nax(Ni-ett)] and p-type poly[Cux(Cu-ett)] [78]. After both polymers were compressed into cuboids, the module composed of 35 p-n couples on an AIN substrate was fabricated with silver and Al to interconnect the bottom and top substrates (Figure 11a,b). The output voltage and short-circuit current increased steadily with temperature, producing an open voltage of 0.26 V and a current of 10.1 mA at ΔT = 80 K (Figure 11c). A maximum output power of 750 μW was generated with a load resistance of 33 Ω under a temperature difference of ΔT = 82 K, which is the highest power ever reported in organic TEGs (Figure 11d). This high TE performance was attributed to the excellent TE properties of both p-type (ZT~0.01) and n-type (ZT~0.1–0.2 around 400 K) conducting polymers in the module.

**Figure 12:**
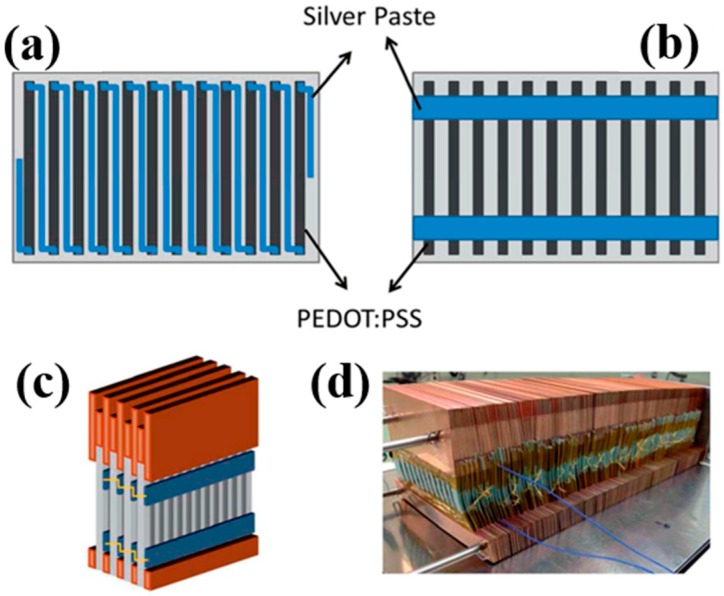
The schematic representation of the (**a**) series and (**b**) parallel PEDOT:PSS arrays; (**c**) the schematic and (**d**) photograph of the PEDOT:PSS modules sandwiched between copper plates. Reprinted with permission from [132]. Copyright 2014 The Royal Society of Chemistry.

Crispin et al. fabricated thermoelectric generators (TEGs) using PEDOT:Tos as a thermoelectric p-type material and TTF-TCNQ as an n-type semiconductor. The maximum power output per area of the PEDOT-Tos/TTF-TCNQ TEGs, consisting of 54 thermocouples with the leg dimensions (25 mm × 25 mm × 30 mm), produced 45 µW·cm^−2^. Wei et al. reported on organic thermoelectric modules screen-printed on paper by using PEDOT:PSS and silver paste, as shown in Figure 12 [132]. They used a highly conductive PEDOT:PSS solutions as an ink to cover a sheet of paper. Silver paste, having the good wettability on PEDOT:PSS, was screen-printed on the PEDOT:PSS legs to create either series (Figure 12a) or parallel (Figure 12b) connections. The large-area devices in which the PEDOT/silver paste arrays were sandwiched between Cu plates (Figure 12c,d) and connected with metal wires either in series or in parallel generated a power output of 50 μW with a temperature gradient of 100 K.

Hybrid nanomaterials in which polymers are combined with carbon nanofillers such as graphene and carbon nanotubes have proven to be promising TE materials in fully utilizing low-graded heat for niche applications. For instance, Dörling et al. demonstrated the TE behavior of polymer composites where an initially p-type polymer/CNT composite was switched to n-type via UV photoinduction [133]. They fabricated flexible TEGs from solution using a single processing step; nitrogen-doped CNTs were dispersed in o-dichlorobenzene and dissolved in P3HT, followed by their mixing, sonicating, and drop-casting on PET substrates. The UV irradiation on the p-type P3HT/MWNT converted it to an n-type composite. A total of 15 double legs of p- and n-type P3HT/MWNT materials, which were attached to a glass filled with ice water, generated a voltage of 5 mV, showing the possibility for application as a wristband.

Wang el al. reported a generator using diethylenetriamine (DETA) as an n-type dopant for SWNTs [134]. They reported TEGs, composed of 14 thermocouples, with a high power output of 649 nW at ΔT = 55 K. Other significant work was published in 2016 by Cho et al. in which the TEGs were fabricated with PANi/Au-doped-CNT (p-type TE materials) and PEI/CNT (n-type TE materials) as TE elements [135]. They achieved 376 nW using 14 thermocouple modules by applying a temperature gradient of 10 K. A unique combination of the layer-by-layer assembly of multiwalled carbon nanotubes and in-situ electrochemical polymerization of PEDOT exhibited high TE properties [136]. The MWNT films (<1 μm thick) infused with PEDOT displayed a power factor of 155 µW/m K^2^. A cylindrical TE module using p-type TE materials of poly(diallyldimethylammonium chloride) (PDDA)/MWNT/PEDOT produced a maximum power of 5.5 µW with a temperature gradient of 30 K.

Recently, textile thermoelectric generators have become very popular in the field of organic TEG due to their easy integration in fabrics for body heat energy recovery. In terms of harvesting low-grade heat generated from the human body, a fabric-based TE material is suitable for wearable self-powered electronic devices with the merit of its flexibility and wearability, which is very difficult to achieve with conventional inorganic-based counterparts. Wu et al. fabricated yarns with a TE functionality in which the yarns were processed with waterborne polyurethane (WPU), MWNT, and PEDOT:PSS composites [137]. Nonionic WPU solution was used as a viscous polymer matrix, and MWNT as a conductive filler was stabilized in PEDOT:PSS in water. The synthesized nonionic WPU was completely dissolved in water because of the hydrophilicity of the PEG in WPU. By increasing the ratio of PEDOT:PSS in MWNT solutions, the electrical conductivity and Seebeck coefficient of the WPU/MWNT-PEDOT:PSS composites increased simultaneously, leading to the enhancement of power factors. At the ratio of 1:4 of MWNT to PEDOT:PSS and doping with 5 wt% DMSO, the composites exhibited a power factor of 1.4 µW∙m^−1^∙K^−2^. Based on the optimized conditions (20 wt% MWNT 1:4 ratios of MWNT to PEDOT:PSS, and doped with 5 wt% DMSO), they applied to typical yarns such as cotton and polyester as the substrate to evaluate the coating feasibility. The resistance of both yarns decreased with the number of dipping cycles, and polyester yarn displayed a lower resistance than that of the cotton, which may stem from a uniform and smooth surface in long continuous filament fibers of the polyester yarn, while the cotton yarn had short fibers with uneven and rough surfaces.

Du et al. made flexible fabric-based TE devices by incorporating PEDOT:PSS onto commercial fabric [138]. The PEDOT:PSS coated fabric maintained the flexibility and softness of the polyester fabric, which displayed a high mechanical compliance upon deformation such as rolling, bending, and twisting. The PEDOT:PSS coated fiber exhibited a power factor of 0.045 µW∙m^−1^∙K^−2^. The electric voltage generated by fabric-based PEDOT:PSS devices increased in a linear relationship with both temperature differences and the number of strips placed in series. The modules of the fabric-based TE generator connected with 5 strips produced 4.3 mV with a ΔT of 75.2 K.

**Figure 13:**
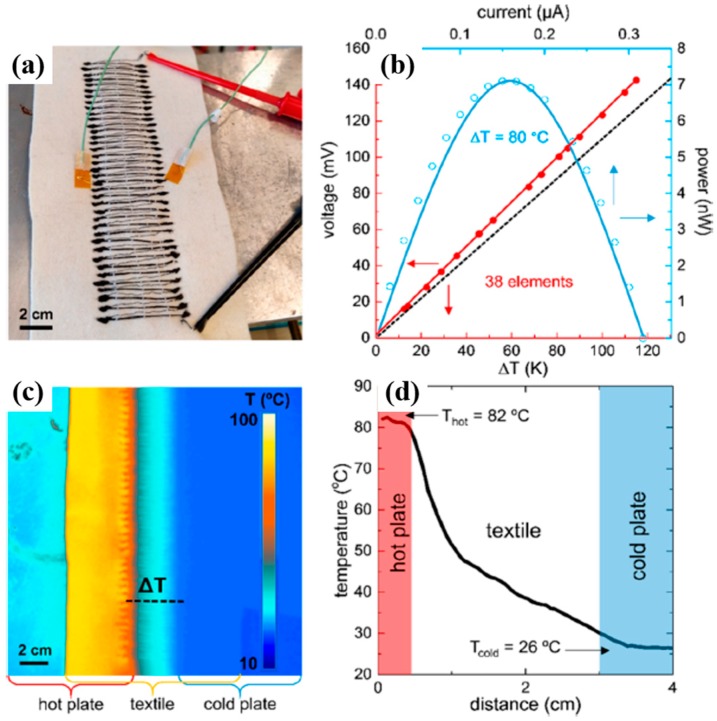
(**a**) The all-organic in-plane embroidered textile thermoelectric (TE) device with 38 n/p elements−constructed with n-type coated PET yarns, p-type dyed silk yarns and a conducting carbon-based paste for electrical connections. (**b**) Electrical measurements of the module with a measured output voltage V_out_ as a function of ΔT (red line), and calculated (dotted line), as well as power output P = V_out_ I as a function of measured current I for ΔT ≈ 80 K. (**c**) Thermal image of the module, placed as in (**a**), with T_hot_ ≈ 82 K and T_cold_ ≈ 26 K. (**d**) Temperature gradient across the textile device. Reprinted with permission from [139]. Copyright 2018 American Chemical Society. Further permissions related to this article should be directed to the American Chemical Society.

A novel design of flexible TE generators, composed of n-type yarns, were fabricated in a way that commercial poly(ethylene terephthalate) (PET) sewing threads were coated with poly(N-vinylpyrrolidine) (PVP)/MWNT nanocomposites [139]. The n-type yarns exhibited an electrical conductivity of 1 S∙cm^−1^ and Seebeck coefficient of −14 µV· K^−1^, which was stable for several months at ambient conditions. A textile TE module of 38 embroidered n/p strips, consisting of the combination of n-type yarns with p-type PEDOT:PSS coated silk yarns, produced an open-circuit voltage of 143 mV with a temperature difference of 116 K and a maximum power output was as high as 7.1 nW at a temperature gradient of 80 K, as shown in Figure 13.

## 5. Summary and Outlook

There is no doubt that in the last few years organic thermoelectric materials have become in a real alternative (in terms of power factor) to the traditional inorganic semiconductors used in the commercial manufacture of thermoelectric generators. Since 2008, the values of the power factors of organic thermoelectric materials have been increased by 4 orders of magnitude while the improvement of inorganic semiconductors has remained in the same order. The possibility of tuning the doping level of organic semiconductors and the control of the polymer chain conformation have been a key to achieving the enormous development of the thermoelectric efficiency of conducting polymers and their corresponding organic composites. Polymer nanocomposites compounded with carbon nanofillers have been shown to rival thermoelectric efficiency in terms of power factor with inorganic-based TE materials; for example, multilayered systems such as PANi/graphene-PEDOT:PSS/PANi/DWNT-PEDOT:PSS (PF ~ 2710 μW·m^−1^·K^−2^) or the annealed CNT web with BV treatment (PF ~ 3100 μW·m^−1^·K^−2^). In addition, carbon materials can behave as n-type semiconductors through doping with molecules or polymers having electron donor groups (such as amine groups). However, there are several challenges to address in the field of organic semiconductors for practical thermoelectric applications. One of the main limitations of organic TE materials is that they cannot be used for high-temperature applications due to their degradation problems. Thus, the temperature gradient across the thermoelectric generator is very limited and the possible way of producing a considerable amount of energy is their use in larger scale areas. For this reason, a future research effort will be needed to find a cost-effective way for producing organic thermoelectric nanocomposites for large-scale areas and to develop a promising technology for their commercial production beyond their simple niche applications. In addition, future investigations should be focused on the integration of organic thermoelectric generators into fabrics in order to harness low-graded energy sources such as body heat, which could be utilized to power small sensors and wireless devices in an environmentally friendly way.

## Figures and Tables

**Table 1:** The chemical structure of polymers used to prepare thermoelectric materials.

Nonconducting Polymers	Abbreviation	Polymer Structure	Conducting Polymers	Abbreviation	Polymer Structure
Polypropylene	PP	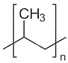	polypyrrole	PPy	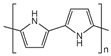
polyvinylpyrrolidone	PVP	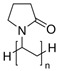	polythiophene	PTh	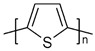
poly(vinyl acetate)	PVAc	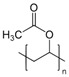	poly(3,4-ethylenedioxythiophene)	PEDOT	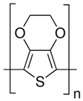
polyethylenimine	PEI	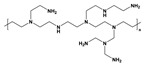	Polyaniline leucoemeraldine(x=1, y=0) emeraldine(x=y=0.5) pernigraniline(x=0, y=1)	PANI	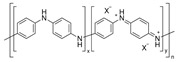
Nafion	Nafion	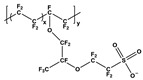	Poly(3,4-ethylenedioxythiophene)-poly(styrenesulfonate)	PEDOT:PSS	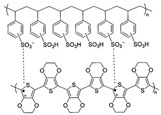

**Table 2:** The thermoelectric properties of polymers and carbon nanofiller-filled polymer composites.

Materials	Systems	Electrical Conductivity (S∙cm^−1^)	Seebeck Coefficient (μV∙K^−1^)	Power Factor (μW∙m^−1^∙K^−2^)	Thermal Conductivity (W·m^−1^∙K^−1^)	ZT	Ref.
Non-Conducting Polymers	PVP	5 × 10^−4^	−9.5	5 × 10^−6^			[25]
	PEI	5.5 × 10^−4^	−5.5	2 × 10^−6^			[25]
Conducting polymers	PANi	173	14	3.5			[26]
		67	12.8	1.1			[27]
		270	20	10.8			[28]
		600			0.276	2.7 × 10^−4^ (423 K)	[29]
	PEDOT:PSS	800	65	400	0.52	0.42 (300 K)	[30]
		1900	20.6	80.63	0.2	0.32	[31]
				318.4	0.3	0.31	[32]
		2980	21.9	142			[33]
	P3HT	0.47	386	7			[34]
		320	269	62.4			[35]
	PPy	4.6	9.4	0.04			[16]
All-Polymer Composites	PEDOT/PEDOT:Tos			446.6		0.44 (300 K)	[31]
	PEDOT:PSS/P3HT	200	17	5.79			[36]
	PEDOT:PSS/PANi-CSA			56			[37]
Graphene-based Composites	PANi/Graphene	1.2 × 10^4^	18.66	4.2	1.2	1.37 × 10^−3^ (393 K)	[38]
			5.6				[39]
		856	19	30.9			[40]
		814	26	55			[41]
		750	28	58.8		4.84 × 10^−4^	[42]
	PEDOT:PSS/Graphene			11		2.1 × 10^−2^	[43]
				624			[44]
		637	26.8	45.7			[45]
				83.2		0.1	[46]
	P3HT/Graphene	1.2	35.5	0.16			[47]
	PPy/Graphene			10.2	0.84	2.8 × 10^−3^	[48]
		75	34	8.6			[49]
	Ppy/Graphene/PANi	500	32	52.5			[50]
CNT-based Composites	Nafion/CNTs	13	28	1			[51]
	PVAc/CNTs	48			0.34	0.006	[52]
	PVAc/SWNT-GA	90.4	40	14.5	0.25		[53]
	PVAc/MWNT-TCPP	1.28	28	0.1			[54]
	PVAc/DWNT-TCPP	71	78	42.8			[55]
	PANi/CNT	61	28.6	5			[56]
		1.6	26	1.1		7.6 × 10^−5^	[57]
		16	10	0.16			[58]
		31.5	45.4	6			[59]
		125	40	20	1.5	0.004	[60]
		1440	39	217			[61]
	PEDOT:PSS/CNT	400	25	25	0.4	0.02 (300 K)	[62]
		1350	41	160			[63]
		960	70	500			[64]
		241	38.9	21.1			[65]
		780	43.7	150			[66]
	P3HT/MWNT	30	28	2.4	0.59	8.7 × 10^−4^	[67]
	P3HT/SWNT			105			[68]
		1000	32.5	107			[69]
		2760	31	308			[70]
	P3HT/CNT	348	97	325			[71]
	PPy/MWNT	72	17	2			[72]
	PPy/SWNT			21.7			[73]
Graphene/CNT-based Composites	PEDOT:PSS/Graphene/MWNT	690	23	37	0.36	0.31 (300 K)	[74]
	PEDT/rGO/SWNT	208	21	9			[75]
	PANi/Graphene/PANi/DWNT	1050	132	1825			[76]
	PANi/Graphene-PEDOT:PSS/PANi/DWNTPEDOT:PSS	1900	120	2710			[77]
n-type TE Materials	poly[Kx (Ni-ett)]			66		0.2	[78]
	PVAc/PEI/CNT-SDBS	15	−100	15			[79]
	PEI/CNT-NaBH_4_	240	−80	153.6			[80]
	DWNT-PEI/Graphene-PVP	300	−80	190			[81]
	PP/SWNT/CuO/PEG	0.024	−57	0.008			[82]
	BV/CNT	2220	−116	3100			[83]

## References

[1] Edenhofer O., Pichs-Madruga R., Sokona Y., Seyboth K., Matschoss P., Kadner S., Zwickel T., Eickemeier P., Hansen G., Schlömer S. (2011). IPCC special report on renewable energy sources and climate change mitigation. Prepared By Working Group III of the Intergovernmental Panel on Climate Change.

[2] Panwar N., Kaushik S., Kothari S. (2011). Role of renewable energy sources in environmental protection: A review. Renew. Sustain. Energy Rev..

[3] Abolhosseini S., Heshmati A., Altmann J. (2013). A Review of Renewable Energy Supply and Energy Efficiency Technologies.

[4] Forman C., Muritala I.K., Pardemann R., Meyer B. (2016). Estimating the global waste heat potential. Renew. Sustain. Energy Rev..

[5] Cullen J.M., Allwood J.M. (2010). Theoretical efficiency limits for energy conversion devices. Energy.

[6] DoE U. (2008). Waste Heat Recovery: Technology and Opportunities in US Industry.

[7] Zhang Q., Sun Y., Xu W., Zhu D. (2014). Organic thermoelectric materials: Emerging green energy materials converting heat to electricity directly and efficiently. Adv. Mater..

[8] Weathers A., Khan Z.U., Brooke R., Evans D., Pettes M.T., Andreasen J.W., Crispin X., Shi L. (2015). Significant Electronic Thermal Transport in the Conducting Polymer Poly(3,4-ethylenedioxythiophene). Adv. Mater..

[9] Vashaee D., Shakouri A. (2004). Improved thermoelectric power factor in metal-based superlattices. Phys. Rev. Lett..

[10] Biswas K., He J., Blum I.D., Wu C.-I., Hogan T.P., Seidman D.N., Dravid V.P., Kanatzidis M.G. (2012). High-performance bulk thermoelectrics with all-scale hierarchical architectures. Nature.

[11] Bahk J.-H., Bian Z., Shakouri A. (2013). Electron energy filtering by a nonplanar potential to enhance the thermoelectric power factor in bulk materials. Phys. Rev. B.

[12] Zhang X., Zhao L.-D. (2015). Thermoelectric materials: Energy conversion between heat and electricity. J. Mater..

[13] Yazdani S., Pettes M.T. (2018). Nanoscale self-assembly of thermoelectric materials: A review of chemistry-based approaches. Nanotechnology.

[14] Russ B., Glaudell A., Urban J.J., Chabinyc M.L., Segalman R.A. (2016). Organic thermoelectric materials for energy harvesting and temperature control. Nat. Rev. Mater..

[15] LeBlanc S. (2014). Thermoelectric generators: Linking material properties and systems engineering for waste heat recovery applications. Sustain. Mater. Technol..

[16] Yue R., Xu J. (2012). Poly(3,4-ethylenedioxythiophene) as promising organic thermoelectric materials: A mini-review. Synth. Metals.

[17] Chen G., Xu W., Zhu D. (2017). Recent advances in organic polymer thermoelectric composites. J. Mater. Chem. C.

[18] Liu J., Wang X., Li D., Coates N.E., Segalman R.A., Cahill D.G. (2015). Thermal conductivity and elastic constants of PEDOT: PSS with high electrical conductivity. Macromolecules.

[19] Blackburn J.L., Ferguson A.J., Cho C., Grunlan J.C. (2018). Carbon-Nanotube-Based Thermoelectric Materials and Devices. Adv. Mater..

[20] Du Y., Xu J., Paul B., Eklund P. (2018). Flexible thermoelectric materials and devices. Appl. Mater. Today.

[21] Shirakawa H., Louis E.J., MacDiarmid A.G., Chiang C.K., Heeger A.J. (1977). Synthesis of electrically conducting organic polymers: Halogen derivatives of polyacetylene, (CH)_x_. J. Chem. Soc. Chem. Commun..

[22] Kaur G., Adhikari R., Cass P., Bown M., Gunatillake P. (2015). Electrically conductive polymers and composites for biomedical applications. RSC Adv..

[23] Pan L., Qiu H., Dou C., Li Y., Pu L., Xu J., Shi Y. (2010). Conducting polymer nanostructures: Template synthesis and applications in energy storage. Int. J. Mol. Sci..

[24] Nguyen D.N., Yoon H. (2016). Recent advances in nanostructured conducting polymers: From synthesis to practical applications. Polymers.

[25] Hiroshige Y., Ookawa M., Toshima N. (2006). High thermoelectric performance of poly(2,5-dimethoxyphenylenevinylene) and its derivatives. Synth. Metals.

[26] Yan H., Toshima N. (1999). Thermoelectric properties of alternatively layered films of polyaniline and (±)-10-camphorsulfonic acid-doped polyaniline. Chem. Lett..

[27] Yan H., Ohta T., Toshima N. (2001). Stretched Polyaniline Films Doped by (±)-10-Camphorsulfonic Acid: Anisotropy and Improvement of Thermoelectric Properties. Macromol. Mater. Eng..

[28] Yao Q., Wang Q., Wang L., Wang Y., Sun J., Zeng H., Jin Z., Huang X., Chen L. (2014). The synergic regulation of conductivity and Seebeck coefficient in pure polyaniline by chemically changing the ordered degree of molecular chains. J. Mater. Chem. A.

[29] Li J., Tang X., Li H., Yan Y., Zhang Q. (2010). Synthesis and thermoelectric properties of hydrochloric acid-doped polyaniline. Synth. Metals.

[30] Kim G.-H., Shao L., Zhang K., Pipe K.P. (2013). Engineered doping of organic semiconductors for enhanced thermoelectric efficiency. Nat. Mater..

[31] Mengistie D.A., Chen C.-H., Boopathi K.M., Pranoto F.W., Li L.-J., Chu C.-W. (2014). Enhanced thermoelectric performance of PEDOT: PSS flexible bulky papers by treatment with secondary dopants. ACS Appl. Mater. Interfaces.

[32] Lee S.H., Park H., Kim S., Son W., Cheong I.W., Kim J.H. (2014). Transparent and flexible organic semiconductor nanofilms with enhanced thermoelectric efficiency. J. Mater. Chem. A.

[33] Wang X., Kyaw A.K.K., Yin C., Wang F., Zhu Q., Tang T., Yee P.I., Xu J. (2018). Enhancement of thermoelectric performance of PEDOT: PSS films by post-treatment with a superacid. RSC Adv..

[34] Zhu H., Liu C., Song H., Xu J., Kong F., Wang J. (2014). Thermoelectric performance of poly(3-hexylthiophene) films doped by iodine vapor with promising high seebeck coefficient. Electron. Mater. Lett..

[35] Qu S., Yao Q., Wang L., Chen Z., Xu K., Zeng H., Shi W., Zhang T., Uher C., Chen L. (2016). Highly anisotropic P3HT films with enhanced thermoelectric performance via organic small molecule epitaxy. NPG Asia Mater..

[36] Shi H., Liu C., Xu J., Song H., Lu B., Jiang F., Zhou W., Zhang G., Jiang Q. (2013). Facile fabrication of PEDOT: PSS/polythiophenes bilayered nanofilms on pure organic electrodes and their thermoelectric performance. ACS Appl. Mater. Interfaces.

[37] Lin-Chung P., Reinecke T. (1995). Thermoelectric figure of merit of composite superlattice systems. Phys. Rev. B.

[38] Wang L., Wang D., Zhu G., Li J., Pan F. (2011). Thermoelectric properties of conducting polyaniline/graphite composites. Mater. Lett..

[39] Du Y., Shen S.Z., Yang W., Donelson R., Cai K., Casey P.S. (2012). Simultaneous increase in conductivity and Seebeck coefficient in a polyaniline/graphene nanosheets thermoelectric nanocomposite. Synth. Metals.

[40] Wang L., Yao Q., Bi H., Huang F., Wang Q., Chen L. (2014). Large thermoelectric power factor in polyaniline/graphene nanocomposite films prepared by solution-assistant dispersing method. J. Mater. Chem. A.

[41] Wang L., Yao Q., Bi H., Huang F., Wang Q., Chen L. (2015). PANI/graphene nanocomposite films with high thermoelectric properties by enhanced molecular ordering. J. Mater. Chem. A.

[42] Zhao Y., Tang G.-S., Yu Z.-Z., Qi J.-S. (2012). The effect of graphite oxide on the thermoelectric properties of polyaniline. Carbon.

[43] Mitra M., Kulsi C., Chatterjee K., Kargupta K., Ganguly S., Banerjee D., Goswami S. (2015). Reduced graphene oxide-polyaniline composites—Synthesis, characterization and optimization for thermoelectric applications. RSC Adv..

[44] Ma W., Liu Y., Yan S., Miao T., Shi S., Xu Z., Zhang X., Gao C. (2018). Chemically doped macroscopic graphene fibers with significantly enhanced thermoelectric properties. Nano Res..

[45] Yoo D., Kim J., Kim J.H. (2014). Direct synthesis of highly conductive poly(3,4-ethylenedioxythiophene): Poly(4-styrenesulfonate)(PEDOT: PSS)/graphene composites and their applications in energy harvesting systems. Nano Res..

[46] Zhang K., Wang S., Zhang X., Zhang Y., Cui Y., Qiu J. (2015). Thermoelectric performance of p-type nanohybrids filled polymer composites. Nano Energy.

[47] Du Y., Cai K., Shen S., Casey P. (2012). Preparation and characterization of graphene nanosheets/poly (3-hexylthiophene) thermoelectric composite materials. Synth. Metals.

[48] Wang L., Liu F., Jin C., Zhang T., Yin Q. (2014). Preparation of polypyrrole/graphene nanosheets composites with enhanced thermoelectric properties. RSC Adv..

[49] Zhang Z., Chen G., Wang H., Zhai W. (2015). Enhanced thermoelectric property by the construction of a nanocomposite 3D interconnected architecture consisting of graphene nanolayers sandwiched by polypyrrole nanowires. J. Mater. Chem. C.

[50] Wang Y., Yang J., Wang L., Du K., Yin Q., Yin Q. (2017). Polypyrrole/graphene/polyaniline ternary nanocomposite with high thermoelectric power factor. ACS Appl. Mater. Interfaces.

[51] Choi Y., Kim Y., Park S.-G., Kim Y.-G., Sung B.J., Jang S.-Y., Kim W. (2011). Effect of the carbon nanotube type on the thermoelectric properties of CNT/Nafion nanocomposites. Org. Electron..

[52] Yu C., Kim Y.S., Kim D., Grunlan J.C. (2008). Thermoelectric behavior of segregated-network polymer nanocomposites. Nano Lett..

[53] Kim Y.S., Kim D., Martin K.J., Yu C., Grunlan J.C. (2010). Influence of Stabilizer Concentration on Transport Behavior and Thermopower of CNT-Filled Latex-Based Composites. Macromol. Mater. Eng..

[54] Moriarty G.P., Wheeler J.N., Yu C., Grunlan J.C. (2012). Increasing the thermoelectric power factor of polymer composites using a semiconducting stabilizer for carbon nanotubes. Carbon.

[55] Hicks L., Dresselhaus M.S. (1993). Thermoelectric figure of merit of a one-dimensional conductor. Phys. Rev. B.

[56] Meng C., Liu C., Fan S. (2010). A promising approach to enhanced thermoelectric properties using carbon nanotube networks. Adv. Mater..

[57] Zhang Q., Wang W., Li J., Zhu J., Wang L., Zhu M., Jiang W. (2013). Preparation and thermoelectric properties of multi-walled carbon nanotube/polyaniline hybrid nanocomposites. J. Mater. Chem. A.

[58] Wang Q., Yao Q., Chang J., Chen L. (2012). Enhanced thermoelectric properties of CNT/PANI composite nanofibers by highly orienting the arrangement of polymer chains. J. Mater. Chem..

[59] Liu J., Sun J., Gao L. (2011). Flexible single-walled carbon nanotubes/polyaniline composite films and their enhanced thermoelectric properties. Nanoscale.

[60] Yao Q., Chen L., Zhang W., Liufu S., Chen X. (2010). Enhanced thermoelectric performance of single-walled carbon nanotubes/polyaniline hybrid nanocomposites. Acs Nano.

[61] Wang L., Yao Q., Xiao J., Zeng K., Qu S., Shi W., Wang Q., Chen L. (2016). Engineered Molecular Chain Ordering in Single-Walled Carbon Nanotubes/Polyaniline Composite Films for High-Performance Organic Thermoelectric Materials. Chemistry.

[62] Kim D., Kim Y., Choi K., Grunlan J.C., Yu C. (2009). Improved thermoelectric behavior of nanotube-filled polymer composites with poly(3,4-ethylenedioxythiophene) poly(styrenesulfonate). ACS Nano.

[63] Yu C., Choi K., Yin L., Grunlan J.C. (2011). Light-weight flexible carbon nanotube based organic composites with large thermoelectric power factors. ACS Nano.

[64] Moriarty G.P., Briggs K., Stevens B., Yu C., Grunlan J.C. (2013). Fully Organic Nanocomposites with High Thermoelectric Power Factors by using a Dual-Stabilizer Preparation. Energy Technol..

[65] Song H., Liu C., Xu J., Jiang Q., Shi H. (2013). Fabrication of a layered nanostructure PEDOT: PSS/SWCNTs composite and its thermoelectric performance. RSC Adv..

[66] Lee W., Kang Y.H., Lee J.Y., Jang K.-S., Cho S.Y. (2016). Improving the thermoelectric power factor of CNT/PEDOT: PSS nanocomposite films by ethylene glycol treatment. RSC Adv..

[67] Wang L., Jia X., Wang D., Zhu G., Li J. (2013). Preparation and thermoelectric properties of polythiophene/multiwalled carbon nanotube composites. Synth. Metals.

[68] Lee W., Hong C.T., Kwon O.H., Yoo Y., Kang Y.H., Lee J.Y., Cho S.Y., Jang K.-S. (2015). Enhanced thermoelectric performance of bar-coated SWCNT/P3HT thin films. ACS Appl. Mater. Interfaces.

[69] Bounioux C., Díaz-Chao P., Campoy-Quiles M., Martín-González M.S., Goni A.R., Yerushalmi-Rozen R., Müller C. (2013). Thermoelectric composites of poly(3-hexylthiophene) and carbon nanotubes with a large power factor. Energy Environ. Sci..

[70] Hong C.T., Lee W., Kang Y.H., Yoo Y., Ryu J., Cho S.Y., Jang K.-S. (2015). Effective doping by spin-coating and enhanced thermoelectric power factors in SWCNT/P3HT hybrid films. J. Mater. Chem. A.

[71] Hong C.T., Kang Y.H., Ryu J., Cho S.Y., Jang K.-S. (2015). Spray-printed CNT/P3HT organic thermoelectric films and power generators. J. Mater. Chem. A.

[72] Wang J., Cai K., Shen S., Yin J. (2014). Preparation and thermoelectric properties of multi-walled carbon nanotubes/polypyrrole composites. Synth. Metals.

[73] Liang L., Chen G., Guo C.-Y. (2016). Enhanced thermoelectric performance by self-assembled layered morphology of polypyrrole nanowire/single-walled carbon nanotube composites. Compos. Sci. Technol..

[74] Yoo D., Kim J., Lee S.H., Cho W., Choi H.H., Kim F.S., Kim J.H. (2015). Effects of one-and two-dimensional carbon hybridization of PEDOT: PSS on the power factor of polymer thermoelectric energy conversion devices. J. Mater. Chem. A.

[75] Li X., Liang L., Yang M., Chen G., Guo C.-Y. (2016). Poly(3,4-ethylenedioxythiophene)/graphene/carbon nanotube ternary composites with improved thermoelectric performance. Org. Electron..

[76] Cho C., Stevens B., Hsu J.H., Bureau R., Hagen D.A., Regev O., Yu C., Grunlan J.C. (2015). Completely organic multilayer thin film with thermoelectric power factor rivaling inorganic tellurides. Adv. Mater..

[77] Cho C., Wallace K.L., Tzeng P., Hsu J.H., Yu C., Grunlan J.C. (2016). Outstanding low temperature thermoelectric power factor from completely organic thin films enabled by multidimensional conjugated nanomaterials. Adv. Energy Mater..

[78] Sun Y., Sheng P., Di C., Jiao F., Xu W., Qiu D., Zhu D. (2012). Organic Thermoelectric Materials and Devices Based on p-and n-Type Poly(metal 1, 1, 2, 2-ethenetetrathiolate)s. Adv. Mater..

[79] Fukumaru T., Fujigaya T., Nakashima N. (2015). Development of n-type cobaltocene-encapsulated carbon nanotubes with remarkable thermoelectric property. Sci. Rep..

[80] Yu C., Murali A., Choi K., Ryu Y. (2012). Air-stable fabric thermoelectric modules made of N-and P-type carbon nanotubes. Energy Environ. Sci..

[81] Cho C., Culebras M., Wallace K.L., Song Y., Holder K., Hsu J.-H., Yu C., Grunlan J.C. (2016). Stable n-type thermoelectric multilayer thin films with high power factor from carbonaceous nanofillers. Nano Energy.

[82] Luo J., Cerretti G., Krause B., Zhang L., Otto T., Jenschke W., Ullrich M., Tremel W., Voit B., Pötschke P. (2017). Polypropylene-based melt mixed composites with singlewalled carbon nanotubes for thermoelectric applications: Switching from p-type to n-type by the addition of polyethylene glycol. Polymer.

[83] An C.J., Kang Y.H., Song H., Jeong Y., Cho S.Y. (2017). High-performance flexible thermoelectric generator by control of electronic structure of directly spun carbon nanotube webs with various molecular dopants. J. Mater. Chem. A.

[84] Yao Q., Chen L., Xu X., Wang C. (2005). The high thermoelectric properties of conducting polyaniline with special submicron-fibre structure. Chem. Lett..

[85] Cao Y., Smith P. (1993). Liquid-crystalline solutions of electrically conducting polyaniline. Polymer.

[86] De Albuquerque J., Melo W., Faria R. (2003). Determination of physical parameters of conducting polymers by photothermal spectroscopies. Rev. Sci. Instrum..

[87] Möller S., Perlov C., Jackson W., Taussig C., Forrest S.R. (2003). A polymer/semiconductor write-once read-many-times memory. Nature.

[88] Bubnova O., Khan Z.U., Malti A., Braun S., Fahlman M., Berggren M., Crispin X. (2011). Optimization of the thermoelectric figure of merit in the conducting polymer poly(3,4-ethylenedioxythiophene). Nat. Mater..

[89] Park T., Park C., Kim B., Shin H., Kim E. (2013). Flexible PEDOT electrodes with large thermoelectric power factors to generate electricity by the touch of fingertips. Energy Environ. Sci..

[90] Scholdt M., Do H., Lang J., Gall A., Colsmann A., Lemmer U., Koenig J.D., Winkler M., Boettner H. (2010). Organic semiconductors for thermoelectric applications. J. Electr. Mater..

[91] Liu C., Lu B., Yan J., Xu J., Yue R., Zhu Z., Zhou S., Hu X., Zhang Z., Chen P. (2010). Highly conducting free-standing poly(3,4-ethylenedioxythiophene)/poly (styrenesulfonate) films with improved thermoelectric performances. Synth. Metals.

[92] Zhang K., Qiu J., Wang S. (2016). Thermoelectric properties of PEDOT nanowire/PEDOT hybrids. Nanoscale.

[93] Ouyang J. (2013). “Secondary doping” methods to significantly enhance the conductivity of PEDOT: PSS for its application as transparent electrode of optoelectronic devices. Displays.

[94] Kim J., Jung J., Lee D., Joo J. (2002). Enhancement of electrical conductivity of poly(3,4-ethylenedioxythiophene)/poly(4-styrenesulfonate) by a change of solvents. Synth. Metals.

[95] Culebras M., Gómez C., Cantarero A. (2014). Enhanced thermoelectric performance of PEDOT with different counter-ions optimized by chemical reduction. J. Mater. Chem. A.

[96] Wang G., Huang W., Eastham N.D., Fabiano S., Manley E.F., Zengg L., Wang B., Zhang X., Chend Z., Lib R. (2017). Aggregation control in natural brush-printed conjugated polymer films and implications for enhancing charge transport. Proc. Natl. Acad. Sci. USA.

[97] Zhu Z., Liu C., Shi H., Jiang Q., Xu J., Jiang F., Xiong J., Liu E. (2015). An effective approach to enhanced thermoelectric properties of PEDOT: PSS films by a DES post-treatment. J. Polym. Sci. Part B Polym. Phys..

[98] Zhang Q., Vigier K.D.O., Royer S., Jerome F. (2012). Deep eutectic solvents: Syntheses, properties and applications. Chem. Soc. Rev..

[99] Kline R.J., McGehee M.D., Kadnikova E.N., Liu J., Fréchet J.M., Toney M.F. (2005). Dependence of regioregular poly (3-hexylthiophene) film morphology and field-effect mobility on molecular weight. Macromolecules.

[100] Teehan S., Efstathiadis H., Haldar P. (2012). Thermoelectric power factor enhancement of AZO/In-AZO quantum well multilayer structures as compared to bulk films. J. Alloys Compounds.

[101] Lee H.J., Anoop G., Lee H.J., Kim C., Park J.-W., Choi J., Kim H., Kim Y.-J., Lee E., Lee S.-G. (2016). Enhanced thermoelectric performance of PEDOT: PSS/PANI–CSA polymer multilayer structures. Energy Environ. Sci..

[102] McGrail B.T., Sehirlioglu A., Pentzer E. (2015). Polymer composites for thermoelectric applications. Angew. Chem. Int. Ed..

[103] Dey A., Bajpai O.P., Sikder A.K., Chattopadhyay S., Khan M.A.S. (2016). Recent advances in CNT/graphene based thermoelectric polymer nanocomposite: A proficient move towards waste energy harvesting. Renew. Sustain. Energy Rev..

[104] Xiang J., Drzal L.T. (2012). Templated growth of polyaniline on exfoliated graphene nanoplatelets (GNP) and its thermoelectric properties. Polymer.

[105] Chatterjee K., Mitra M., Kargupta K., Ganguly S., Banerjee D. (2013). Synthesis, characterization and enhanced thermoelectric performance of structurally ordered cable-like novel polyaniline–bismuth telluride nanocomposite. Nanotechnology.

[106] Kim G.H., Hwang D.H., Woo S.I. (2012). Thermoelectric properties of nanocomposite thin films prepared with poly(3, 4-ethylenedioxythiophene) poly(styrenesulfonate) and graphene. Phys. Chem. Chem. Phys..

[107] Vavro J., Llaguno M.C., Satishkumar B., Luzzi D.E., Fischer J.E. (2002). Electrical and thermal properties of C 60-filled single-wall carbon nanotubes. Appl. Phys. Lett..

[108] Zhang K., Zhang Y., Wang S. (2013). Enhancing thermoelectric properties of organic composites through hierarchical nanostructures. Sci. Rep..

[109] Paloheimo J., Isotalo H., Kastner J., Kuzmany H. (1993). Conduction mechanisms in undoped thin films of C60 and C60/70. Synth. Metals.

[110] Lu Y., Song Y., Wang F. (2013). Thermoelectric properties of graphene nanosheets-modified polyaniline hybrid nanocomposites by an in situ chemical polymerization. Mater. Chem. Phys..

[111] Han S., Zhai W., Chen G., Wang X. (2014). Morphology and thermoelectric properties of graphene nanosheets enwrapped with polypyrrole. RSC Adv..

[112] Dresselhaus M., Dresselhaus G., Hofmann M. (2008). Other one-dimensional systems and thermal properties. J. Vacuum Sci. Technol. B Microelectron. Nanometer Struct. Proc. Meas. Phenom..

[113] Botiz I., Stingelin N. (2014). Influence of molecular conformations and microstructure on the optoelectronic properties of conjugated polymers. Materials.

[114] See K.C., Feser J.P., Chen C.E., Majumdar A., Urban J.J., Segalman R.A. (2010). Water-processable polymer− nanocrystal hybrids for thermoelectrics. Nano Lett..

[115] Zhang Z., Chen G., Wang H., Li X. (2015). Template-Directed In Situ Polymerization Preparation of Nanocomposites of PEDOT: PSS-Coated Multi-Walled Carbon Nanotubes with Enhanced Thermoelectric Property. Chemistry.

[116] Lin Y.-J., Ni W.-S., Lee J.-Y. (2015). Effect of incorporation of ethylene glycol into PEDOT: PSS on electron phonon coupling and conductivity. J. Appl. Phys..

[117] Rivadulla F., Mateo-Mateo C., Correa-Duarte M. (2010). Layer-by-layer polymer coating of carbon nanotubes: Tuning of electrical conductivity in random networks. J. Am. Chem. Soc..

[118] Liu J., Qiu L., Portale G., Koopmans M., Ten Brink G., Hummelen J.C., Koster L.J.A. (2017). N-Type Organic Thermoelectrics: Improved Power Factor by Tailoring Host–Dopant Miscibility. Adv. Mater..

[119] Hwang S., Potscavage W.J., Yang Y.S., Park I.S., Matsushima T., Adachi C. (2016). Solution-processed organic thermoelectric materials exhibiting doping-concentration-dependent polarity. Phys. Chem. Chem. Phys..

[120] Collins P.G., Bradley K., Ishigami M., Zettl D.A. (2000). Extreme oxygen sensitivity of electronic properties of carbon nanotubes. Science.

[121] Wan C., Gu X., Dang F., Itoh T., Wang Y., Sasaki H., Kondo M., Koga K., Yabuki K., Snyder G.J. (2015). Flexible n-type thermoelectric materials by organic intercalation of layered transition metal dichalcogenide TiS_2_. Nat. Mater..

[122] Watts P.C., Mureau N., Tang Z., Miyajima Y., Carey J.D., Silva S.R.P. (2007). The importance of oxygen-containing defects on carbon nanotubes for the detection of polar and non-polar vapours through hydrogen bond formation. Nanotechnology.

[123] Yoo D., Lee J.J., Park C., Choi H.H., Kim J.-H. (2016). N-type organic thermoelectric materials based on polyaniline doped with the aprotic ionic liquid 1-ethyl-3-methylimidazolium ethyl sulfate. RSC Adv..

[124] Zuo G., Li Z., Wang E., Kemerink M. (2018). High Seebeck Coefficient and Power Factor in n-Type Organic Thermoelectrics. Adv. Electron. Mater..

[125] Hewitt C.A., Kaiser A.B., Roth S., Craps M., Czerw R., Carroll D.L. (2012). Multilayered carbon nanotube/polymer composite based thermoelectric fabrics. Nano Lett..

[126] Nonoguchi Y., Nakano M., Murayama T., Hagino H., Hama S., Miyazaki K., Matsubara R., Nakamura M., Kawai T. (2016). Simple Salt-Coordinated n-Type Nanocarbon Materials Stable in Air. Adv. Funct. Mater..

[127] Ryu Y., Freeman D., Yu C. (2011). High electrical conductivity and n-type thermopower from double-/single-wall carbon nanotubes by manipulating charge interactions between nanotubes and organic/inorganic nanomaterials. Carbon.

[128] Freeman D.D., Choi K., Yu C. (2012). N-type thermoelectric performance of functionalized carbon nanotube-filled polymer composites. PLoS ONE.

[129] Snyder G.J., Toberer E.S. (2011). Complex thermoelectric materials. Materials For Sustainable Energy: A Collection of Peer-Reviewed Research and Review Articles from Nature Publishing Group.

[130] Zhao L.-D., Lo S.-H., Zhang Y., Sun H., Tan G., Uher C., Wolverton C., Dravid V.P., Kanatzidis M.G. (2014). Ultralow thermal conductivity and high thermoelectric figure of merit in SnSe crystals. Nature.

[131] Kishore R., Priya S. (2018). A Review on Low-Grade Thermal Energy Harvesting: Materials, Methods and Devices. Materials.

[132] Wei Q., Mukaida M., Kirihara K., Naitoh Y., Ishida T. (2014). Polymer thermoelectric modules screen-printed on paper. RSC Adv..

[133] Dörling B., Ryan J.D., Craddock J.D., Sorrentino A., Basaty A.E., Gomez A., Garriga M., Pereiro E., Anthony J.E., Weisenberger M.C. (2016). Photoinduced p-to n-type Switching in Thermoelectric Polymer-Carbon Nanotube Composites. Adv. Mater..

[134] Wu G., Gao C., Chen G., Wang X., Wang H. (2016). High-performance organic thermoelectric modules based on flexible films of a novel n-type single-walled carbon nanotube. J. Mater. Chem. A.

[135] An C.J., Kang Y.H., Lee A.-Y., Jang K.-S., Jeong Y., Cho S.Y. (2016). Foldable thermoelectric materials: Improvement of the thermoelectric performance of directly spun CNT webs by individual control of electrical and thermal conductivity. ACS Appl. Mater. Interfaces.

[136] Culebras M., Cho C., Krecker M., Smith R., Song Y., Gómez C.M., Cantarero A.S., Grunlan J.C. (2017). High thermoelectric power factor organic thin films through combination of nanotube multilayer assembly and electrochemical polymerization. ACS Appl. Mater. Interfaces.

[137] Wu Q., Hu J. (2016). Waterborne polyurethane based thermoelectric composites and their application potential in wearable thermoelectric textiles. Compo. Part B Eng..

[138] Du Y., Cai K., Chen S., Wang H., Shen S.Z., Donelson R., Lin T. (2015). Thermoelectric fabrics: Toward power generating clothing. Sci. Rep..

[139] Ryan J.D., Lund A., Hofmann A.I., Kroon R., Sarabia-Riquelme R., Weisenberger M.C., Müller C. (2018). All-Organic Textile Thermoelectrics with Carbon-Nanotube-Coated n-Type Yarns. ACS Appl. Energy Mater..

